# Elimination of methicillin-resistant *Staphylococcus aureus* biofilms on titanium implants via photothermally-triggered nitric oxide and immunotherapy for enhanced osseointegration

**DOI:** 10.1186/s40779-023-00454-y

**Published:** 2023-05-04

**Authors:** Yong-Lin Yu, Jun-Jie Wu, Chuan-Chuan Lin, Xian Qin, Franklin R. Tay, Li Miao, Bai-Long Tao, Yang Jiao

**Affiliations:** 1 Department of Pathology, Affiliated Hospital of Zunyi Medical University, Zunyi, 563003 Guizhou China; 2 Laboratory Research Center, the First Affiliated Hospital of Chongqing Medical University, Chongqing, 400016 China; 3 Department of Blood Transfusion, Laboratory of Radiation Biology, the Second Affiliated Hospital, Army Military Medical University, Chongqing, 400037 China; 4 Department of Reproductive Endocrinology, Chongqing Health Center for Women and Children, Chongqing, 401147 China; 5 The Graduate School, Augusta University, Augusta, GA 30912 USA; 6 Department of Stomatology, the Seventh Medical Center of PLA General Hospital, Beijing, 100700 China

**Keywords:** Polydopamine nanoparticles, Methicillin-resistant *Staphylococcus aureus*, Nitric oxide, Osseointegration, Osteo-immunomodulation, Photothermal effect, Titanium implants

## Abstract

**Background:**

Treatment of methicillin-resistant *Staphylococcus aureus* (MRSA) biofilm infections in implant placement surgery is limited by the lack of antimicrobial activity of titanium (Ti) implants. There is a need to explore more effective approaches for the treatment of MRSA biofilm infections.

**Methods:**

Herein, an interfacial functionalization strategy is proposed by the integration of mesoporous polydopamine nanoparticles (PDA), nitric oxide (NO) release donor sodium nitroprusside (SNP) and osteogenic growth peptide (OGP) onto Ti implants, denoted as Ti-PDA@SNP-OGP. The physical and chemical properties of Ti-PDA@SNP-OGP were assessed by scanning electron microscopy, X-ray photoelectron spectroscope, water contact angle, photothermal property and NO release behavior. The synergistic antibacterial effect and elimination of the MRSA biofilms were evaluated by 2′,7′-dichlorofluorescein diacetate probe, 1-N-phenylnaphthylamine assay, adenosine triphosphate intensity, o-nitrophenyl-β-d-galactopyranoside hydrolysis activity, bicinchoninic acid leakage. Fluorescence staining, assays for alkaline phosphatase activity, collagen secretion and extracellular matrix mineralization, quantitative real‑time reverse transcription‑polymerase chain reaction, and enzyme-linked immunosorbent assay (ELISA) were used to evaluate the inflammatory response and osteogenic ability in bone marrow stromal cells (MSCs), RAW264.7 cells and their co-culture system. Giemsa staining, ELISA, micro-CT, hematoxylin and eosin, Masson’s trichrome and immunohistochemistry staining were used to evaluate the eradication of MRSA biofilms, inhibition of inflammatory response, and promotion of osseointegration of Ti-PDA@SNP-OGP in vivo.

**Results:**

Ti-PDA@SNP-OGP displayed a synergistic photothermal and NO-dependent antibacterial effect against MRSA following near-infrared light irradiation, and effectively eliminated the formed MRSA biofilms by inducing reactive oxygen species (ROS)-mediated oxidative stress, destroying bacterial membrane integrity and causing leakage of intracellular components (*P* < 0.01). In vitro experiments revealed that Ti-PDA@SNP-OGP not only facilitated osteogenic differentiation of MSCs, but also promoted the polarization of pro-inflammatory M1 macrophages to the anti-inflammatory M2-phenotype (*P* < 0.05 or *P* < 0.01). The favorable osteo-immune microenvironment further facilitated osteogenesis of MSCs and the anti-inflammation of RAW264.7 cells via multiple paracrine signaling pathways (*P* < 0.01). In vivo evaluation confirmed the aforementioned results and revealed that Ti-PDA@SNP-OGP induced ameliorative osseointegration in an MRSA-infected femoral defect implantation model (*P* < 0.01).

**Conclusions:**

These findings suggest that Ti-PDA@SNP-OGP is a promising multi-functional material for the high-efficient treatment of MRSA infections in implant replacement surgeries.

**Supplementary Information:**

The online version contains supplementary material available at 10.1186/s40779-023-00454-y.

## Background

Biomaterial-associated infection (BAI) is a global health burden that is responsible for approximately 40% of all hospital-acquired infections in the USA [1]. Infections occur throughout the service life of implants, but not only during the implantation process, which inevitably result in the failure of titanium (Ti) implants [2, 3]. The formation of methicillin-resistant *Staphylococcus aureus* (MRSA) biofilms on the surfaces of Ti implants exacerbates the burden of BAI [4]. The extracellular polymeric substances (EPS) are presented in the matrix secreted by MRSA, which can protect the intramembranous bacteria from the host’s immune system and external environmental challenges such as the permeation of antibiotics and environmental pressure [5, 6]. Removal and replacement of the infected implants is often the Hobson’s choice in the management of implant-associated MRSA infections because of the ineffectiveness of conventional antibiotics in eliminating MRSA biofilms [7, 8]. The less than ideal postoperative osseointegration capability of Ti implants further reduces their effectiveness [9]. Therefore, there is a need to develope a novel strategy that simultaneously eliminates the MRSA biofilms and improves the osseointegration of Ti implants without inducing drug resistance.

Photothermal therapy (PTT) is a non-intrusive approach that has been extensively explored to eliminate biofilms. This approach is characterized by deep tissue penetration, application adaptability, high selectivity, low risks for drug resistance and minimal side effects [10–12]. Commonly used photothermal agents include metal nanoparticles (e.g., gold and copper nanoparticles), organic molecules (e.g., porphyrin, indocyanine green, thiadiazol derivatives), carbon-based materials (e.g.*,* graphene oxide, carbon nanotubes, carbon nitride) and metal sulfides (e.g., copper sulfide, cuprous sulfide, molybdenum disulfide) [13–17]. Polydopamine nanoparticles (PDA) are promising photothermal agents because of their high photothermal conversion ability, excellent biocompatibility and feasibility for modification [18–20]. In a previous study, composite hydrogels comprising of lauric acid-grafted chitosan, dibenzaldehyde-modified polyethylene glycol and curcumin-loaded PDA nanoparticles were reported to exhibit potent antibacterial ability against wound infections. This activity was attributed to the synergistic effect between near-infrared light (NIR)-triggered, the on-demand release of curcumin as well as hyperthermia [21]. However, the biofilms were only eradicated by the application of NIR irradiation at 70 °C. Although the human body can endure relatively high local temperatures for a short time, the surrounding normal tissues may be damaged under high-temperature conditions [22, 23]. The mild temperature (~ 5 °C) induced by PTT results in less adverse effects, but the antibacterial and anti-biofilm activities are significantly reduced at that mild temperature [24, 25]. To address this issue, scientists have combined PTT with the application of antibacterial agents for the inhibition of biofilm formation and elimination of established biofilms.

The combination of gas therapy with PTT is an auspicious approach to improve the effectiveness of PTT against bacterial infection, infected wounds, inflammation, cardiovascular diseases, and cancers [26–28]. Treatment of bacterial infections by gas therapy involves the use of a high volume of gaseous molecules such as hydrogen, hydrogen sulfide, carbon monoxide, carbon dioxide, sulfur dioxide, and nitric oxide (NO) [29–34]. NO is an important endogenous gas molecule in physiological and pathological processes. The antibacterial effectiveness of NO is attributed to its damaging effect on proteins and DNA molecules at high concentrations. NO possesses potent antibacterial activity against bacterial invasion in mammals without causing drug resistance [35, 36]. Nevertheless, the use of gas therapy for the treatment of bacterial infections is limited by insufficient gas accumulation at the infected site, uncontrolled release behavior and imprecise therapeutic mechanisms [37, 38]. To date, different NO-triggering approaches have been investigated. These approaches include glutathione, enzyme, pH, H_2_O_2_, and photothermal treatments. Among them, PTT is a promising and efficient approach because of its deep tissue penetration and controllable release behavior of NO under NIR irradiation [23]. Consequently, there is a pressing need to develop NIR laser-stimulated drug delivery systems with an “on-demand” release behavior to improve the specificity of gas therapy.

With the development of nanotechnology, diazeniumdiolates (NONOates), N,N′-disecbutyl-N,N′-dinitroso-p-phenylenediamine (BNN6), S-nitrosoglutathione (GSNO), and L-arginine (L-Arg) have been widely employed as NO donors [23, 28]. However, issues such as by-product toxicity, short half-life, insufficient gas accumulation, uncontrolled release behavior and imprecise therapeutic mechanisms weaken the therapeutic efficacy of these compounds in vivo [28]. In comparison, sodium nitroprusside (SNP) is more biocompatible than others and may be used as a photothermally-triggered donor for the “on-demand” release of NO, due to its sensitivity to high temperatures [39, 40].

In this study, in order to achieve the eradication of MRSA biofilms and enhanced osseointegration, a multi-functional strategy was proposed by coupling NO gas therapy, PTT with peptide-drug therapy onto Ti implants.

## Methods

### Materials

Ti rods (length: 10 mm, diameter: 15 mm) and Ti foils (10 mm × 10 mm, 0.25 mm thick) were obtained from the Northwest Institute for Non-ferrous Metal Research (Xi’an, China). Dopamine hydrochloride, fluorescein diacetate, propidium iodide, Pluronic F-127, DCFH-DA, o-nitrophenyl-β-D-galactopyranoside (ONPG) and Hoechst 33258 were purchased from MilliporeSigma (St. Louis, MO, USA). SNP, tris(hydroxymethyl) animomethane (Tris), 1,3,5-trimethylbenzene, polymyxin B, and N-phenyl-1-naphthylamine were purchased from Aladdin Industrial Co. Ltd. (Shanghai, China). Cell counting kit-8 (CCK-8), Mueller Hinton broth (MHB), agarose and paraformaldehyde were obtained from Solarbio Biotechnology Co. (Beijing, China). Bicinchoninic acid (BCA) assay kit, Griess reagent, lactate dehydrogenase (LDH) assay kit, BCIP/NBT alkaline phosphatase (ALP) staining kit, ALP assay kit, Sirius red staining kit, Alizarin red sodium salt, enhanced adenosine triphosphate (ATP) assay kit, 3,3′-diaminobenzidine (DAB) detection kit, hematoxylin and eosin (HE) staining kit, Masson’s trichrome staining kit and Giemsa staining kit were purchased from Beyotime Biotechnology Co. (Jiangsu, China). Osteogenic growth peptide (OGP, ALKRQGRTLYGFGG) was purchased from Top‐Peptide Co., Ltd. (Shanghai, China). Rhodamine-phalloidin, Trizol reagent, and primers were purchased from Invitrogen Co. (CA, USA). Enzyme-linked immunosorbent assay (ELISA) kits for Runt-related transcription factor-2 (Runx2), bone morphogenetic protein-2 (BMP2), vascular endothelial growth factor (VEGF), transforming growth factor-β (TGF-β), tumor necrosis factor-α (TNF-α), interleukin-6 (IL-6) and IL-10 were purchased from ABclonal Biotechnology. Co., Ltd. (Wuhan, China). Other chemicals were purchased from Xingguang Chemical Co. (Chongqing, China).

### Synthesis of PDA, PDA@SNP and PDA@SNP-OGP nanoparticles

Pluronic F-127 (0.36 g) and 1,3,5-trimethylbenzene (0.36 g) were mixed and dissolved in H_2_O (65 ml) and ethanol (60 ml) mixture. Tris (90 mg) and dopamine hydrochloride (60 mg) were added and stirred for 24 h under dark condition. The template and PDA were eliminated by centrifugation. The PDA was washed three times with the mixture of ethanol and acetone. The synthesized PDA nanoparticles were dispersed in ethanol and stored at -20 °C for subsequent analysis.

PDA@SNP nanoparticles were prepared by dispersing PDA (5 mg) nanoparticles in ethanol. A pre-prepared SNP solution (2.5 mg/ml) was introduced under stirring condition. The black mixture was collected by centrifugation (11,000 r/min, 10 min) after 24 h and the PDA@SNP nanoparticles were rinsed with deionized water.

The PDA@SNP nanoparticles were immersed in Tris–HCl buffer (10 mmol/L, pH 8.5) containing OGP (2 mg/ml) to ensure covalent immobilization of OGPs. After the mixture was stirred for 24 h, the PDA@SNP-OGP nanoparticles were collected and rinsed with deionized water. The morphology of PDA, PDA@SNP and PDA@SNP-OGP was analyzed by transmission electron microscopy (TEM, Talos F200S, ThermoFisher Scientific, Waltham, MA, USA). The total amount of OGP conjugated on the PDA@SNP nanoparticles was determined by ultraviolet–visible (UV–Vis) spectrophotometer (UV-3600, Shimadzu, Japan).

### Preparation of Ti-PDA@SNP-OGP substrate

Clean Ti foils were soaked in dopamine hydrochloride solution (2 mg/ml) containing 10 mmol/L Tris buffer (20 ml, pH 8.5) and incubated for 24 h. PDA, PDA@SNP or PDA@SNP-OGP nanoparticles (0.3 mg) were subsequently immobilized on Ti substrate through the dopamine coating. The specimens were rinsed with deionized water and denoted as Ti-PDA, Ti-PDA@SNP or Ti-PDA@SNP-OGP. The morphology, surface chemistry, and water contact angles (WCA) of these substrates were analyzed by scanning electron microscopy (SEM, Quattro S, ThermoFisher Scientific, Waltham, MA, USA), X-ray photoelectron spectroscope (XPS, Empyrean, Netherlands) and contact angle goniometry (SDC-200S, Sindin, China), respectively. The cross-sectional image and thickness of the PDA@SNP-OGP coating on the Ti implant were observed by SEM. The coating adhesion strength was investigated using a scratch tester (CSM Instruments, Switzerland).

### Photothermal effect of PDA, PDA@SNP, and PDA@SNP-OGP nanoparticles

The photothermal effect of the prepared nanoparticles was evaluated using NIR radiation emitted from an 808 nm laser (Mild-River Company, China). Real-time temperature changes were recorded using a digital thermometer (HH806AU, Omega Engineering, Norwalk, CT, USA). Briefly, PDA, PDA@SNP and PDA@SNP-OGP nanoparticles (0.5 mg/ml) were exposed to 808 nm laser (1.00 W/cm^2^) for 10 min. The photothermal conversion efficacy (*η*), heating and cooling curves of PDA, PDA@SNP, and PDA@SNP-OGP nanoparticles (0.5 mg/ml) were evaluated according to the following equations:

1 $$\eta =\frac{hS\left({T}_{max}-{T}_{sur}\right)-{Q}_{0}}{I(1-{10}^{-A808})}$$

2 $$\uptau_{{\text{s}}} = \left( {m_{d} {\text{c}}_{d} } \right)/\left( {hS} \right)$$

3 $$Q_{0} = hS\left( {T_{max,\,water} - T_{surr} } \right)$$

where *T*_*max*_ is the maximum temperature induced by the specimens (PDA, PDA@SNP or PDA@SNP-OGP nanoparticles), *T*_*max,water*_ is the maximum temperature of water, *T*_*surr*_ is the ambient room temperature. *Q*_*0*_ is the background energy input without specimens and calculated from Eq. (3). *I* is the laser power (1 W/cm^2^), *A808* is the absorbance of sample (PDA, PDA@SNP and PDA@SNP-OGP nanoparticles) at 808 nm, *h* is the heat transfer coefficient, *S* is the sample container surface area, *m*_*d*_ is the weight of PDA, PDA@SNP or PDA@SNP-OGP nanoparticles solution, and *c*_*d*_ is the heat capacity of water.

### Photothermal effect of Ti or functionalized Ti substrate

The photothermal effect of native Ti or functionalized Ti substrate was evaluated using an infrared thermal imaging system (E40 IR image system, FLIR, Wilsonville, OR, USA). Ti was soaked in phosphate-buffered saline (PBS) buffer (500 μl) and exposed to NIR irradiation (1.00 W/cm^2^, 10 min). Temperature changes of each specimen were recorded and the time interval was set as 30 s. In addition, temperature changes of the Ti-PDA@SNP-OGP irradiated with different NIR power densities (0.25, 0.50, 1.00, and 1.25 W/cm^2^) were evaluated.

### Detection of NIR radiation-stimulated NO release

Griess reagent was used to determine the NO level. Ti-PDA@SNP-OGP was exposed to NIR (1.00 W/cm^2^) for 10 min, followed by a 20 min without NIR irradiation, and this procedure was repeated 5 times. The resultant medium was treated with Griess reagent (50 µl). The amount of NO released from Ti-PDA@SNP-OGP was determined at 548 nm using a UV–Vis spectrophotometer. The cumulative release profiles of NO from Ti, Ti-PDA, Ti-PDA@SNP, and Ti-PDA@SNP-OGP were also evaluated. The concentration of released NO was calculated using a standard curve.

### In vitro antibacterial activity

MRSA (ATCC33591) was used to evaluate the anti-biofilm property. The specimens were cultured with 1 ml MRSA suspension [1 × 10^8^ colony forming unit (CFU)/ml] in the stationary growth phase at 37 °C for 3 d, and the MHB culture medium was replaced every day. The anti-biofilm property and mechanism were determined using the spread plate assay, SEM examination, reactive oxygen species (ROS) generation, membrane permeability, ATP intensity, and ONPG hydrolysis with or without NIR irradiation.

#### Spread plate assay

For anti-biofilm activity, the specimens were cultured with 1 ml MRSA suspension (1 × 10^8^ CFU/ml) in the stationary growth phase and incubated at 37 °C for 3 d. The specimens were then exposed to NIR irradiation (1.00 W/cm^2^) for 10 min. Non-attached bacteria were detached by gently washing with PBS. One milliliter of sterile PBS was added to each well and the treated MRSA was removed from the substrates through ultrasonication for 10 min. The bacterial suspension was diluted 10,000-fold with sterile PBS. Then, 100 μl of diluted bacterial suspension was spread onto the agar plates. The CFUs were imaged and counted. The antibacterial ratio of each substrate was calculated using the formula: A = (B – C)/B × 100%; where A is the antibacterial ratio, B is the average CFU of the control group (Ti) and C is the average CFU of the experimental group.

#### SEM

The MRSA biofilm was cultured on Ti or functionalized Ti substrate with or without NIR irradiation for 10 min. The biofilms were stripped by ultrasound to generate a bacterial suspension. The suspension was added to a silicon wafer and fixed overnight with paraformaldehyde (4 wt%) at 4 °C. The specimens were dehydrated with an ascending ethanol series (25%, 50%, 75% and 100%) and further dehydrated with tert-butanol for 10 min. The dried specimens were sputtered-coated with gold for SEM examination.

#### ROS generation

ROS generation in the different groups was evaluated using 2′,7′-dichlorofluorescein diacetate (DCFH-DA) probe. An MRSA suspension (1 ml, 1 × 10^6^ CFU/ml) was cultured on Ti, Ti-PDA, Ti-PDA@SNP or Ti-PDA@SNP-OGP, with/without 808 nm NIR laser irradiation. The bacterial suspension was incubated for 24 h and treated with DCFH-DA solution (10 μm). After incubating the specimens for 30 min, the fluorescence intensity was determined using a fluorescence spectrophotometer (RF5301PC, Shimadzu, Kyoto, Japan) with an excitation wavelength of 488 nm and an emission wavelength of 525 nm.

#### Membrane permeability

A 1-N-phenylnaphthylamine (NPN) fluorescent probe method was used to examine the membrane permeability of MRSA. An MRSA suspension was obtained by centrifugation (5000 r/min, 8 min) and treated with the NPN fluorescent probe (10 μl, 10 μmol/L) for 30 min. The fluorescence intensity of the solution was determined using a fluorescence spectrophotometer (excitation wavelength 350 nm, emission wavelength 420 nm). An MRSA suspension treated with polymyxin B was used as the positive control. The fluorescence intensities of the experimental groups were normalized to that of the control group (Ti without NIR irradiation).

#### BCA leakage

The BCA assay was performed to investigate the protein leakage of MRSA after various treatments. MRSA (1 ml, 1 × 10^6^ CFU/ml) was seeded over Ti, Ti-PDA, Ti-MPDA@SNP, or Ti-PDA@SNP-OGP. After that, the culture medium was collected, and vortexed for 20 s. Then, the mixture (400 μl) was taken out, filtered with a syringe filter (0.22 μm). Next, the filtrate sample (25 μl) was added to the standard BCA protein assay kit and incubated at 37 °C with gentle shaking (200 r/min). Lastly, the absorbance of the obtained solution was determined at 490 nm using a spectrophotometric microplate reader (Bio-Rad 680, Hercules, CA, USA). The protein leakage amount = (the protein amount in each experimental group − the protein amount in control group). LB medium with Ti, Ti-PDA, Ti-PDA@SNP, and Ti-PDA@SNP-OGP without adding MRSA was used as the control group, and LB medium with Ti, Ti-PDA, Ti-PDA@SNP, and Ti-PDA@SNP-OGP with adding MRSA was used as the experimental groups.

#### ATP intensity

An ATP assay kit was used to evaluate the ATP intensity of MRSA under different conditions. MRSA (1 ml, 1 × 10^6^ CFU/ml) was seeded over Ti, Ti-PDA, Ti-MPDA@SNP or Ti-PDA@SNP-OGP. Half of the specimens from each group were exposed to NIR irradiation while the other half were not irradiated. The ATP intensities were evaluated using a fluorescence spectrophotometer at 562 nm. The relative fluorescence intensity of the experimental groups was obtained by normalizing the fluorescence intensity to that of pristine Ti without NIR irradiation (control group).

#### ONPG hydrolysis

ONPG hydrolysis was conducted to evaluate the membrane permeability of bacteria presented within the MRSA biofilms. The MRSA biofilms were treated with/without 808 NIR irradiation for 10 min. The biofilms grown on the different substrates were harvested by sonication (10 min) and incubated with ONPG solution (500 μl, 0.75 mol/L). The optical density values of the supernatants were determined at 405 nm using a spectrophotometric microplate reader (Bio-Rad 680, Hercules, CA, USA).

#### Bacterial enzymatic activity evaluation

For the respiratory chain dehydrogenase activity, MRSA (1 ml, 1 × 10^6^ CFU/ml) was incubated with Ti, Ti-PDA, Ti-PDA@SNP, and Ti-PDA@SNP-OGP with/without laser irradiation at 37 °C for 12 h. Then, the cultured medium (250 μl), glucose solution (1 ml, 100 mmol/L), tris (hydro xymethyl) aminomethane (TRIS) buffer (1 ml, 50 mmol/l, pH = 8.6), 4% 2,3,5-triphenyltetrazolium chloride (TTC) solution, and LB broth (250 μl) were added into a tube. After incubating for 6 h, concentrated sulfuric acid (50 μl) was added into the above mixture to stop the reaction. Afterward, the formed enzymatic reaction product [1,3,5-triphenylformazan (TPF)] was extracted with toluene. Lastly, the absorbance of obtained solution was determined at 490 nm using a spectrophotometric microplate reader (Bio-Rad 680, Hercules, CA, USA). The relative activity of respiratory chain dehydrogenase (%) was calculated according to the following formula: A = B/C × 100%. Where A indicates the relative activity of respiratory chain dehydrogenase; B is the average OD_490_ value of control (Ti without NIR irradiation); and C is the average OD_490_ value of experimental samples.

#### Leakage of intracellular components

The leakage of intracellular components as an indicator for the evaluation of membrane integrity in bacteria was measured by OD_260_ approach. 1 ml of MRSA (5 × 10^8^ CFU/ml) was cultured with Ti, Ti-PDA, Ti-PDA@SNP, and Ti-PDA@SNP-OGP substrates. Half of the specimens from each group were exposed to NIR irradiation while the other half were not irradiated. After that, the mixture was filtered with a syringe filter (0.22 μm) to remove the bacteria and other materials. Lastly, the absorbance of obtained solution was determined at 260 nm by an UV–Vis spectrophotometer (UV-3600, Shimadzu, Japan).

### Cell culture

Bone marrow stromal cells (MSCs) were isolated from the tibiae and femur of ten Sprague–Dawley (SD) rats (male, 100–120 g) as previously reported [41–43]. The SD rats were provided by Chongqing Medical University and the number of production license for experimental animals was SCXK 2022-0010. The medium was changed every 2 d and MSCs from the third passage were used in the subsequent experiments. RAW264.7 cells (Army Medical University, Chongqing, China) were cultured in high glucose Dulbecco’s Modified Eagle’s Medium (DMEM) supplemented with streptomycin/penicillin, and 10% (v/v) fetal bovine serum (Hyclone, USA) under 5% CO_2_ at 37 °C.

#### Cell adhesion, morphology, and proliferation

Bone MSCs (1 × 10^4^ cells/well) or RAW264.7 cells (1 × 10^4^ cells/well) were incubated with different substrates for 2 d [20]. The cells were then lysed with Triton X-100 (0.2%) for 5 min and treated with a rhodamine-phalloidin solution overnight for cytoskeleton staining. After staining, the specimens were rinsed with PBS and the MSCs or RAW264.7 cells nuclei were stained with Hoechst 33258 (200 μl).

Morphological changes in MSCs or RAW264.7 cells were evaluated using a confocal laser scanning microscopy (CLSM; FV3000, Olympus, Tokyo, Japan).

After incubation for 1, 4, or 7 d, a mixture of CCK-8 solution and culture medium (1:9, v/v) was added to the wells of each group. Optical density values of the supernatants were determined after incubation for 2 h using a spectrophotometric microplate reader at 450 nm. RAW264.7 cells were cultured onto different substrates for 1, 3 and 5 d. The CCK-8 assay was performed to evaluate cell proliferation.

#### Osteoblastic differentiation of MSCs

Cells cultured on different substrates for 7 d were analyzed using an ALP kit. Bone MSCs cultured on different substrates were stained with Sirius red solution for 2 h. The stained cells were treated with NaOH to dissolve the red crystals. The optical density values of supernatants were measured at 540 nm using a spectrophotometric microplate reader.

Additional MSCs were incubated for 21 d and stained with Alizarin red (0.1%) to evaluate the mineralization level of the extracellular matrix (ECM).

The stained mineral nodules were dissolved with CH_3_COOH solution (10%) and the optical density values of supernatants were determined at 405 nm using a microplate reader.

#### In vitro anti-inflammation evaluation

RAW264.7 cells (5 × 10^4^ cells/ml) were activated by adding lipopolysaccharide (LPS) solution (40 ng/ml) into the medium to stimulate polarization of macrophages into M1-type, followed by co-incubation with different substrates. After culturing for 24 h, the anti-inflammatory activity was evaluated based on the expression levels of representative genes of M1-type [*CD86,* inducible nitric oxide synthase (*iNOS*) and *CD11C*], M2-type [*CD206*, arginase-1 (*Arg-1*) and *CD163*], pro-inflammatory cytokines (*TNF-α* and *IL-1β*), and anti-inflammatory cytokines (*IL-10* and *IL-1ra*).

##### Quantitative real‑time reverse transcription-polymerase chain reaction (qRT-PCR)

Bone MSCs or RAW264.7 cells were cultured in 24-well plates with different substrates. The total RNA was extracted from MSCs or RAW264.7 cells using Total RNA kit (Qiagen, Hilden, Germany). The RNA was reversely transcribed into complementary DNA using a reverse transcription kit (Takara, Shiga, Japan). A Bio-Rad CFX Manager system was used for qRT-PCR, and the primers used are listed in Additional file 1: Table S1. Relative levels of gene expression were obtained by normalizing the expression levels to the level of the housekeeping gene *β-actin*.

##### ELISA

The concentrations of TNF-α, IL-1β, IL-10, and IL-6 secreted by RAW264.7 cells seeded on different substrates were determined using ELISA kits. RAW264.7 cells (5 × 10^4^ cells/ml) were cultured on different substrates and incubated for 3 d. The samples were centrifuged, the supernatants were collected, and cytokine concentrations were determined using standard curves.

#### MSC migration

A Transwell co-culture experiment was performed to evaluate the osteogenic differentiation of MSCs under the influence of RAW264.7 cells. Bone MSCs (1 × 10^4^ cells/well) were seeded in the upper Transwell chamber (Corning, New York, USA; pore size: 8 μm, inner diameter: 6.5 mm), and co-cultured with RAW264.7 cells for 24 h [41]. The MSCs that migrated through the membrane were fixed with 4% paraformaldehyde and stained with 1% crystal violet reagent. The stained MSCs were analyzed using an inverted microscope (Zeiss, Jena, Germany).

#### Cell interaction

RAW264.7 cells (1 × 10^4^ cells/well) were cultured on Ti or functionalized Ti substrate for 2 d. The specimen-conditioned medium was collected and centrifuged (1000 r/min, 4 min) to remove residual cells. The MSCs were then cultured on 24-well plates with addition of the conditional medium (1 ml) from each group. The specimen-conditioned medium was changed every 2 d. The cells were treated with 5-bromo-4-chloro-3-indolyl phosphate (BCIP)/nitro blue tetrazolium (NBT), Sirius red and Alizarin red S staining reagents at a predetermined time. The mRNA expression levels of osteogenesis-related genes (*Runx2*, *BMP2*, *ALP*, *OPN* and *OCN*) were evaluated.

### Implantation surgery

All in vivo animal experiments were conducted according to the institutional guidelines and relevant regulations for Animal Experimentation of Laboratory Animals of Chongqing Medical University and the Seventh Medical Center of PLA General Hospital, and approved by the Animal Ethics Committees of the Chongqing Medical University (2021-738) and the Seventh Medical Center of PLA General Hospital (2021-110). Forty SD rats (male, 200–250 g) were provided from Chongqing Medical University and used for implantation surgery. The rats were anesthetized and the surgical area was shaved and disinfected. An MRSA-infected femoral defect model was successfully constructed by making a cylindrical defect (1.5 mm diameter) using a surgical drill at the center of the femoral condyle in the direction of the medullary cavity. The surgical sites were sutured after gently inserting the prepared Ti implants with formed MRSA biofilms into the bone defects. The implantation site was exposed to NIR irradiation (1.00 W/cm^2^) for 10 min, and real-time temperature changes were recorded using a thermal camera.

#### Evaluation of anti-biofilm property and inflammatory response

Rats were sacrificed to collect the femur samples 3 d after implantation, and the embedded implants were gently removed. The implants were soaked in MHB and sonicated to strip off the adhered MRSA. The bacterial suspension was cultured for 12 h, diluted (10,000 times) and inoculated on MHB agar plates. The anti-biofilm efficacy of each specimen was investigated. The bacteria colonies on the plates were evaluated and photographed after culturing at 37 °C for 24 h. The implants were soaked in MHB medium and cultured for 14 h. The turbidity of each group was photographed and the turbidity level was determined. Meanwhile, ELISA was performed to determine the TNF-α, IL-6, TGF-β and IL-10 concentrations. Besides, HE and immunohistochemistry (IHC) staining were performed using anti-CD68 and anti-CD206 antibodies to investigate the osteo-immunomodulatory effect [42]. Bone tissue sections were dewaxed, hydrated in a descending series of ethanol (100–50%), antigen retrieved, blocked for 30 min and incubated with primary antibodies (CD86 and CD206) overnight at 4 °C. The sections were then treated with the corresponding horseradish peroxidase-conjugated secondary antibodies at room temperature for 1 h, then stained with a DAB detection kit for color reaction and counterstained with hematoxylin.

#### Bone formation and biosafety evaluation in vivo

Rats were sacrificed 30 d after implantation to examine the extent of osseointegration between the implants and original bone tissues. Analyses were conducted using micro-computed tomography (micro-CT; Viva CT40, SCANCO Medical AG, Brüttisellen, Switzerland), HE, and Masson’s trichrome staining. For micro-CT, the harvested femurs were fixed with formalin reagent and incubated for 2 d. The treated femurs were scanned with micro-CT and analyzed as previously described [42]. For HE and Masson’s trichrome staining, the implants were gently removed from the femurs and stained. The stained specimens were observed using an inverted microscope (Zeiss). Biosafety was also evaluated using whole-blood biochemical analysis.

### Statistical analysis

All data were presented as means ± standard deviation (SD). Data were processed in Origin software (version 8.0) by Tukey’s test via one-way analysis of variance (ANOVA) for statistical analysis. Statistical significance was pre-set at α = 0.05.

## Results

### Synthesis and characterization of PDA@SNP-OGP nanoparticles

**Fig. 1:**
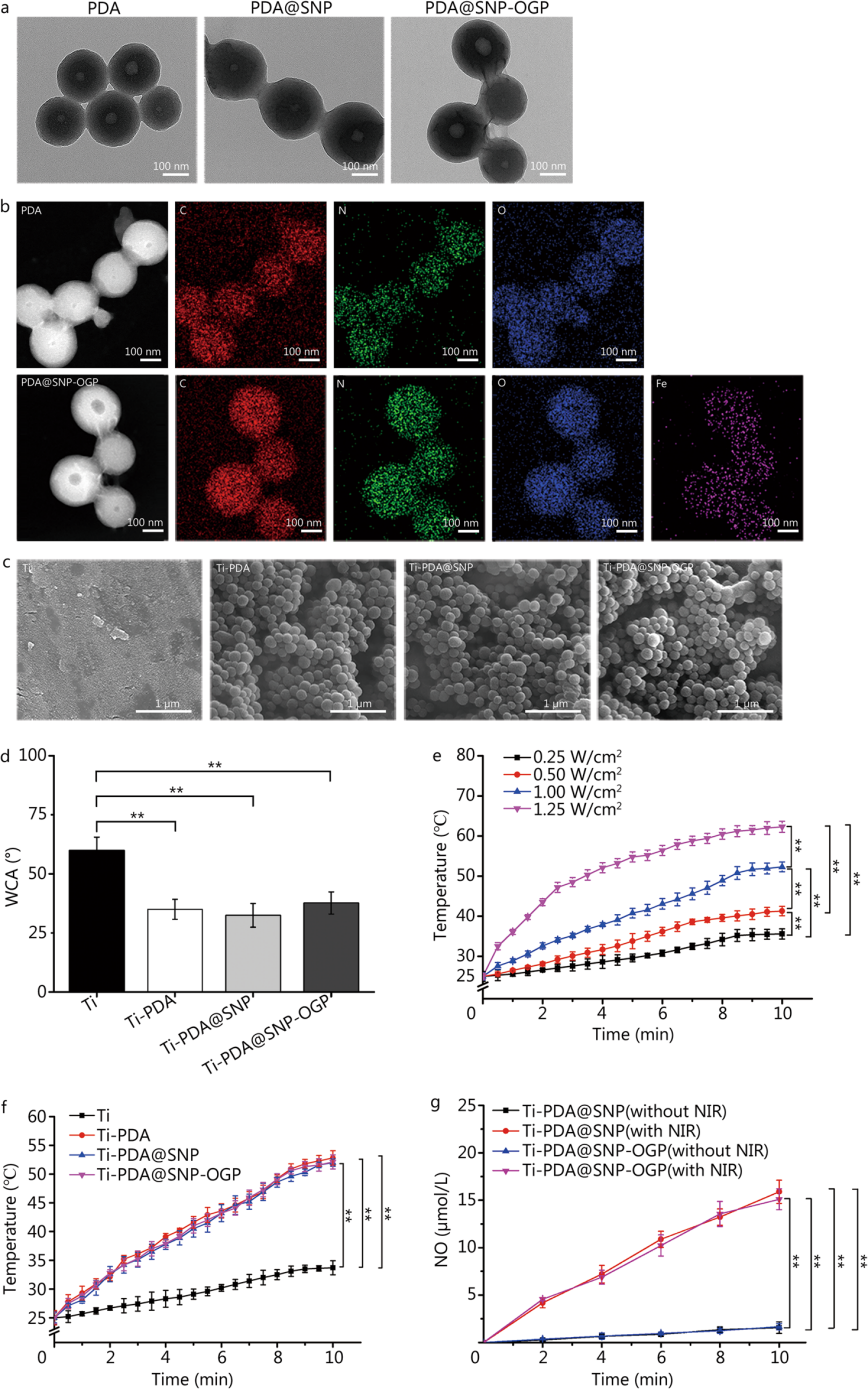
Characterization of Ti-PDA@SNP-OGP. **a** Transmission electron microscopy (TEM) images of PDA, PDA@SNP or PDA@SNP-OGP nanoparticles. Scale bar = 100 nm. **b** Elemental mappings of PDA and PDA@SNP-OGP nanoparticles. Scale bar = 100 nm. **c** Scanning electron microscopy (SEM) images of Ti, Ti-PDA, Ti-PDA@SNP or Ti-PDA@SNP-OGP. Scale bar = 1 μm. **d** Water contact angles (WCA) of Ti, Ti-PDA, Ti-PDA@SNP and Ti-PDA@SNP-OGP. **e** Heating curves of Ti-PDA@SNP-OGP using NIR irradiation with different power intensities. **f** Heating curves of Ti, Ti-PDA, Ti-PDA@SNP or Ti-PDA@SNP-OGP with NIR irradiation (808 nm, 1.00 W/cm^2^). **g** The cumulative concentrations of NO from Ti-PDA@SNP or Ti-PDA@SNP-OGP with or without NIR irradiation (808 nm, 1.00 W/cm^2^) for 10 min. **P < 0.01; Ti titanium, PDA polydopamine nanoparticles, SNP sodium nitroprusside, OGP osteogenic growth peptide, NO nitric oxide, NIR near-infrared light

As shown in Fig. 1a, PDA showed well-dispersed spherical morphology with an average diameter of (176 ± 12) nm. The average diameters of PDA@SNP and PDA@SNP-OGP were slightly increased to (183 ± 18) nm and (196 ± 21) nm after loading of SNP and OGP modification, respectively. TEM elemental mapping showed that iron (Fe) was uniformly distributed in PDA@SNP-OGP (Fig. 1b). Differential light scattering showed that the size of PDA@SNP-OGP was relatively larger than the sizes of PDA and PDA@SNP (Additional file 1: Fig. S1a), which is consistent with TEM images. According to Fourier Transform infrared spectroscopy (FTIR) results, the absorption bands at 1125 and 840 cm^−1^ were attributed to υ-NH and υ-Ar of the benzene ring. Absorption peaks of PDA@SNP at 2102 and 1885 cm^−1^ corresponded to υ-CN (axial and equatorial CN ligands) and υ-NO, indicating that SNP was successfully loaded onto PDA. The υ-CN and υ-NO bands of SNP, and the υ-NH_2_ and υ-CONH bands of OGP were observed after modification with OGP (Additional file 1: Fig. S1b). Based on the pre-prepared standard curve [40], the SNP loading rate of PDA@SNP was 8.92%—using the UV–Vis light spectroscopy spectra (Additional file 1: Fig. S1c).

### Preparation and characterization of Ti-PDA@SNP-OGP substrate

SEM was used to examine the morphologies of Ti or functionalized Ti substrate. Ti had a relatively smooth surface (Fig. 1c). The PDA, PDA@SNP, and PDA@SNP-OGP were uniformly distributed on Ti with no obvious difference in morphology. Elemental mappings of the distribution of Ti, O, C, N and Fe in Ti-PDA@SNP-OGP. XPS analysis of Ti identified signals of Ti (88.92%) and O (11.08%). As for Ti-PDA@SNP-OGP, three new peaks (C, N, and Fe) were identified (Additional file 1: Fig. S1d). The C 1 s spectra of Ti-PDA@SNP-OGP showed that the main peaks were concentrated at the following locations: C=O (287.7 eV), C−O (286.3 eV), C−N (285.4 eV), C−C (284.6 eV), and C=C (284.1 eV). Two O 1 s peaks at 532.9 and 531.0 eV were attributed to O−C and O=C. In the N 1 s high-resolution spectrum of Ti-PDA@SNP-OGP, four sub-peaks at 399.8, 399.2, 398.9, and 398.2 eV were assigned to –NH_2_, –N=O, –N=C, N−C, respectively (Additional file 1: Fig. S1e). These results are indicative of the modification made by OGP on PDA@SNP-OGP. Ti was more hydrophobic with a WCA of (60.0 ± 5.6)°. The WCA of Ti-PDA, Ti-PDA@SNP and Ti-PDA@SNP-OGP were (35.0 ± 4.2)°, (32.4 ± 5.0)° and (37.7 ± 4.8)°, respectively (Fig. 1d). It was showed the cross-sectional SEM image of a PDA@SNP-OGP coating on Ti. The thickness of the coating on Ti ranged from 3.7 to 8.3 μm (Additional file 1: Fig. S2a). The adhesion strength of PDA@SNP-OGP coatings on Ti was measured using a scratch tester (CSM Instruments, Switzerland). The critical loads (Lc1 and Lc2) of Ti-PDA@SNP-OGP were about 1.14 and 1.26 N, respectively (Additional file 1: Fig. S2b). The data suggested good mechanical stability of PDA@SNP-OGP coating after it was applied to Ti. This observation was consistent with the result of a previous study [4].

### Photothermal effect and NO release of Ti-PDA@SNP-OGP substrate

The digital NIR photothermal imaging system was used to evaluate the photothermal effect of Ti-PDA@SNP-OGP at different NIR power intensities. The temperature of Ti-PDA@SNP-OGP was 35.6 °C at 0.25 W/cm^2^, 41.3 °C at 0.50 W/cm^2^, 52.3 °C at 1.00 W/cm^2^, and 62.3 °C at 1.25 W/cm^2^ after irradiation for 10 min (Fig. 1e). Temperature changes were further evaluated for Ti or functionalized Ti substrate using irradiation power (1.00 W/cm^2^). The temperature of Ti increased from 25.0 to 33.7 °C after irradiation for 10 min. However, the temperatures for Ti-PDA, Ti-PDA@SNP and Ti-PDA@SNP-OGP increased to 52.8, 52.0, and 52.1 °C, respectively (Fig. 1f). The photothermal conversion efficacy (*η*) of Ti-PDA@SNP-OGP was found to be 20.3% after exposure to NIR irradiation (Additional file 1: Fig. S2c). The morphology of Ti-PDA@SNP-OGP did not change after NIR irradiation (1.00 W/cm^2^, 10 min) (Additional file 1: Fig. S2d).

The cumulative concentrations of NO released from Ti-PDA@SNP and Ti-PDA@SNP-OGP were (15.9 ± 1.2) μmol/L and (15.1 ± 1.1) μmol/L after NIR irradiation after 10 min, respectively. However, the cumulative concentration of NO released from Ti-PDA@SNP or Ti-PDA@SNP-OGP did not increase without NIR irradiation (Fig. 1g). The cumulative concentration of NO released from NIR-irradiated Ti-PDA@SNP-OGP was significantly increased (*P* < 0.01, Additional file 1: Fig. S2e), reaching (34.6 ± 2.4) μmol/L after 30 min (Additional file 1: Fig. S2f), which generates a NIR-triggered “on–off” switch mode for NO release (Additional file 1: Fig. S2g). However, the cumulative concentration of NO released from the Ti-PDA@SNP-OGP after incubation for 72 h was only (4.9 ± 0.8) μmol/L without NIR irradiation (Additional file 1: Fig. S2h).

### Inhibition and eradication of MRSA biofilms

**Fig. 2:**
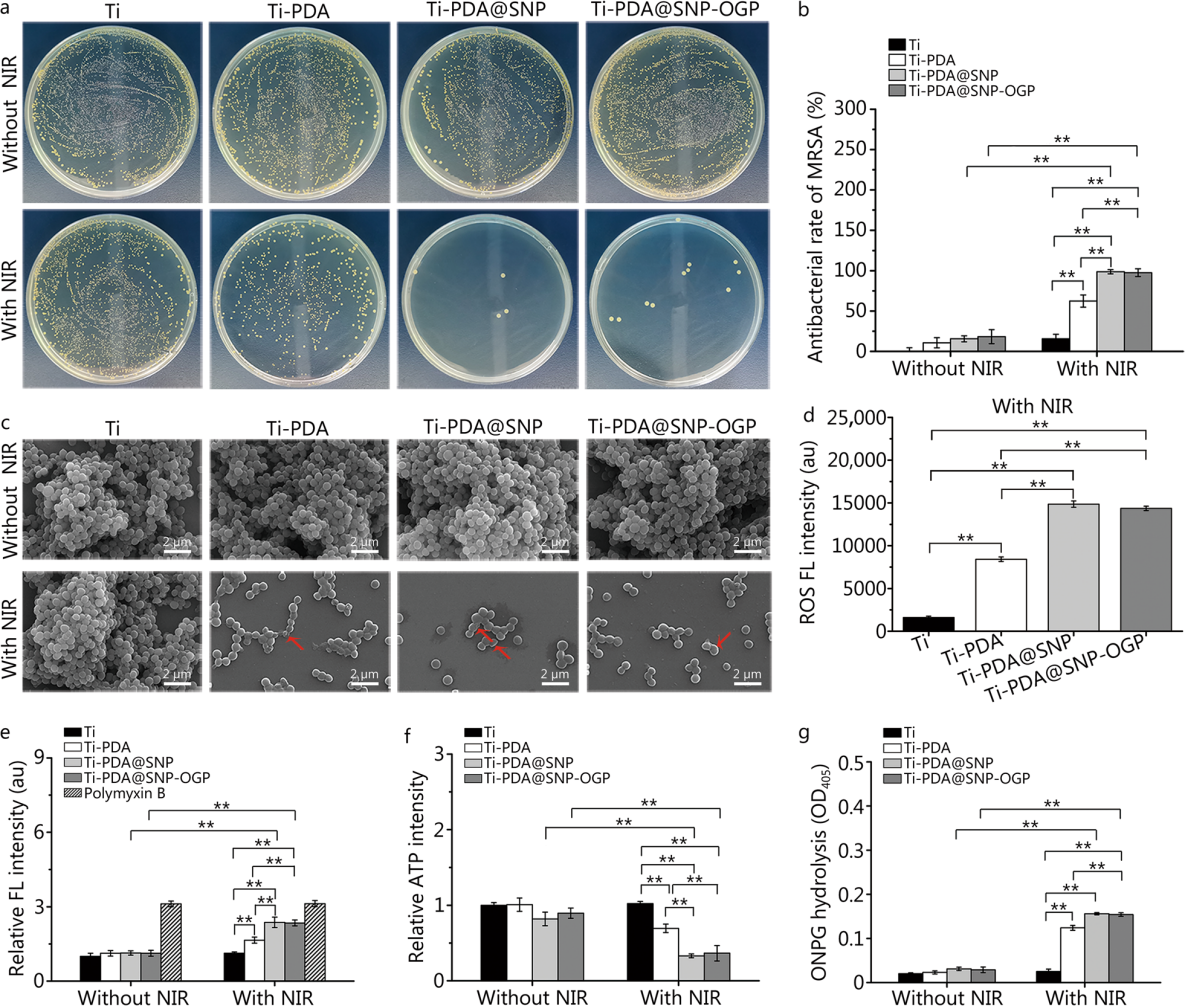
In vitro anti-biofilm activity of Ti-PDA@SNP-OGP with or without NIR irradiation. **a**. Representative images of MRSA colonies in agar plates from each group with or without NIR irradiation. **b** Elimination efficacy of MRSA biofilms based on the results of agar plates in each group. **c** Scanning electron microscopy (SEM) images of MRSA biofilms from each group withor without NIR irradiation. Scale bar = 2 μm. Red arrows indicate the damaged cell membranes. **d** ROS FL intensity of MRSA from each group by DCFH-DA probe under NIR irradiation. **e** Relative FL intensity of MRSA under various conditions by NPN fluorescent probe, polymyxin B was used as the positive control. The fluorescence intensities of the experimental groups were normalized to that of pristine Ti without NIR irradiation. **f** Relative ATP intensity of MRSA in each group measured with a fluorescence spectrophotometer. The relative fluorescence intensity of the experimental groups was obtained by normalizing the fluorescence intensity to that of pristine Ti without NIR irradiation. **g** ONPG hydrolysis of MRSA from each group using a spectrophotometric microplate reader. ^**^P < 0.01; Ti titanium, PDA polydopamine nanoparticles, SNP sodium nitroprusside, OGP osteogenic growth peptide, NIR near-infrared light, MRSA methicillin-resistant *Staphylococcus aureus* scanning, ROS reactive oxygen species, DCFH-DA 2′,7′-dichlorofluorescein diacetate, NPN 1-N-phenylnaphthylamine, FL fluorescence, ATP adenosine triphosphate, ONPG o-nitrophenyl-β-D-galactopyranoside

A dilution spread plating approach was used for investigating MRSA biofilm inhibition and eradication on Ti or functionalized Ti substrate with or without NIR irradiation. All substrates demonstrated no obvious bacteriostatic properties against MRSA colonies without NIR irradiation. On the contrary, the number of MRSA colonies formed on the agar plates in Ti-PDA@SNP or Ti-PDA@SNP-OGP was markedly decreased compared with that of Ti after NIR irradiation (Fig. 2a). After 10 min of NIR irradiation, the elimination rates of MRSA biofilms by Ti-PDA, Ti-PDA@SNP and Ti-PDA@SNP-OGP were (62.4 ± 7.6)%, (98.7 ± 2.6)%, and (97.6 ± 4.7)%, respectively (Fig. 2b). Based on SEM images, the MRSA exhibited a spherical and integrated membrane structure on Ti, Ti-PDA, Ti-PDA@SNP or Ti-PDA@SNP-OGP without NIR irradiation. After NIR irradiation, MRSA biofilms were partially destroyed in Ti-PDA, with withered and damaged (indicated by red arrows) cell membranes. After NIR irradiation, the morphology of MRSA biofilms on Ti was still smooth and compact. In contrast, MRSA biofilms were eliminated and the majority of bacteria were dead with non-focusable margins on Ti-PDA@SNP or Ti-PDA@SNP-OGP (Fig. 2c).

The MRSA biofilms were stained dark purple on Ti, Ti-PDA, Ti-PDA@SNP or Ti-PDA@SNP-OGP without NIR irradiation (Additional file 1: Fig. S3a). After NIR irradiation, staining on Ti-PDA@SNP and Ti-PDA@SNP-OGP became brighter, and biofilm biomass was 0.31 and 0.37, respectively (*P* < 0.01, Additional file 1: Fig. S3b). The biofilm biomass decreased to 68.9% and 72.3% after 2 min of NIR irradiation on Ti-PDA@SNP and Ti-PDA@SNP-OGP, respectively. When MRSA biofilms were irradiated for longer periods (over 10 min), the biofilm biomass on Ti-PDA@SNP and Ti-PDA@SNP-OGP was reduced to 8.1% and 13.3%, respectively (Additional file 1: Fig. S3c). The anti-biofilm capability of antibiotic vancomycin was further compared with the effects achieved using Ti or NIR-irradiated Ti-PDA@SNP-OGP. The MRSA viability of vancomycin was 29.6%, which was significantly higher than that of NIR-irradiated Ti-PDA@SNP-OGP (8.3%) (*P* < 0.01, Additional file 1: Fig. S3d). Furthermore, the relative biofilm biomass in NIR-irradiated Ti-PDA@SNP-OGP was significantly lower (10.4%) than that of Ti (100.0%) and vancomycin (95.6%, *P* < 0.01, Additional file 1: Fig. S3e). Collectively, these results indicate that hyperthermia and photothermally-triggered NO release by Ti-PDA@SNP-OGP displayed a synergistic antibacterial effect against MRSA and could effectively eradicate MRSA biofilms.

### Mechanism of inhibition on MRSA biofilms

The ROS level was investigated using a DCFH-DA probe. After NIR irradiation, ROS levels are much higher in Ti-PDA@SNP or Ti-PDA@SNP-OGP, compared with Ti or Ti-PDA (*P* < 0.01; Fig. 2d). By comparison, all groups had similar ROS levels in the absence of NIR irradiation (Additional file 1: Fig. S3f). Without NIR irradiation, the relative FL intensity of Ti-PDA, Ti-PDA@SNP, and Ti-PDA@SNP-OGP was not obviously increased as compared to Ti group. Under laser irradiation, the relative FL intensities of Ti-PDA@SNP and Ti-PDA@SNP-OGP were significantly enhanced, which were similar to positive control (*P* < 0.01, Fig. 2e). Compared with Ti, Ti-PDA@SNP-OGP had a more pronounced decline in ATP intensity (~63.5%) (Fig. 2f) and a more exhaustive extent of BCA leakage (~2.6 times) after NIR irradiation (Additional file 1: Fig. S3g). In contrast, there was only a 30.5% decrease in ATP intensity (Fig. 2f) and a 1.75 times increase in BCA leakage in NIR-irradiated Ti-PDA (Additional file 1: Fig. S3g). Hydrolysis of ONPG was used to examine the extent of bacterial membrane damage (i.e., the extent of ONPG hydrolysis increases when bacterial membranes are impaired). Without NIR irradiation, the differences in the extent of ONPG hydrolysis in Ti-PDA, Ti-PDA@SNP, and Ti-PDA@SNP-OGP were negligible when compared with Ti. After NIR irradiation, the extent of ONPG hydrolysis in Ti-PDA@SNP or Ti-PDA@SNP-OGP was significantly higher than that in Ti or Ti-PDA (*P* < 0.01, Fig. 2g). Furthermore, respiratory chain dehydrogenase activity was measured after different treatments. Respiratory chain dehydrogenase was inactivated in Ti-PDA@SNP or Ti-PDA@SNP-OGP after NIR irradiation (*P* < 0.01, Additional file 1: Fig. S3h). Alterations in bacterial membrane permeability are usually associated with the leakage of intracellular components such as deoxyribonucleic acid (DNA) or ribonucleic acid (RNA). MRSA biofilms in Ti-PDA@SNP-OGP demonstrated a remarkably high concentration of the leakage of intracellular components after NIR irradiation (*P* < 0.05 or *P* < 0.01, Additional file 1: Fig. S3i), which is consistent with the result of ONPG hydrolysis. Collectively, these results indicate that ROS-mediated oxidative stress, destruction of bacterial membrane integrity and leakage of intracellular components were the main factors of bacterial death and eradication of MRSA biofilms induced by Ti-PDA@SNP-OGP.

### In vitro cytocompatibility and osteogenic differentiation potential

**Fig. 3:**
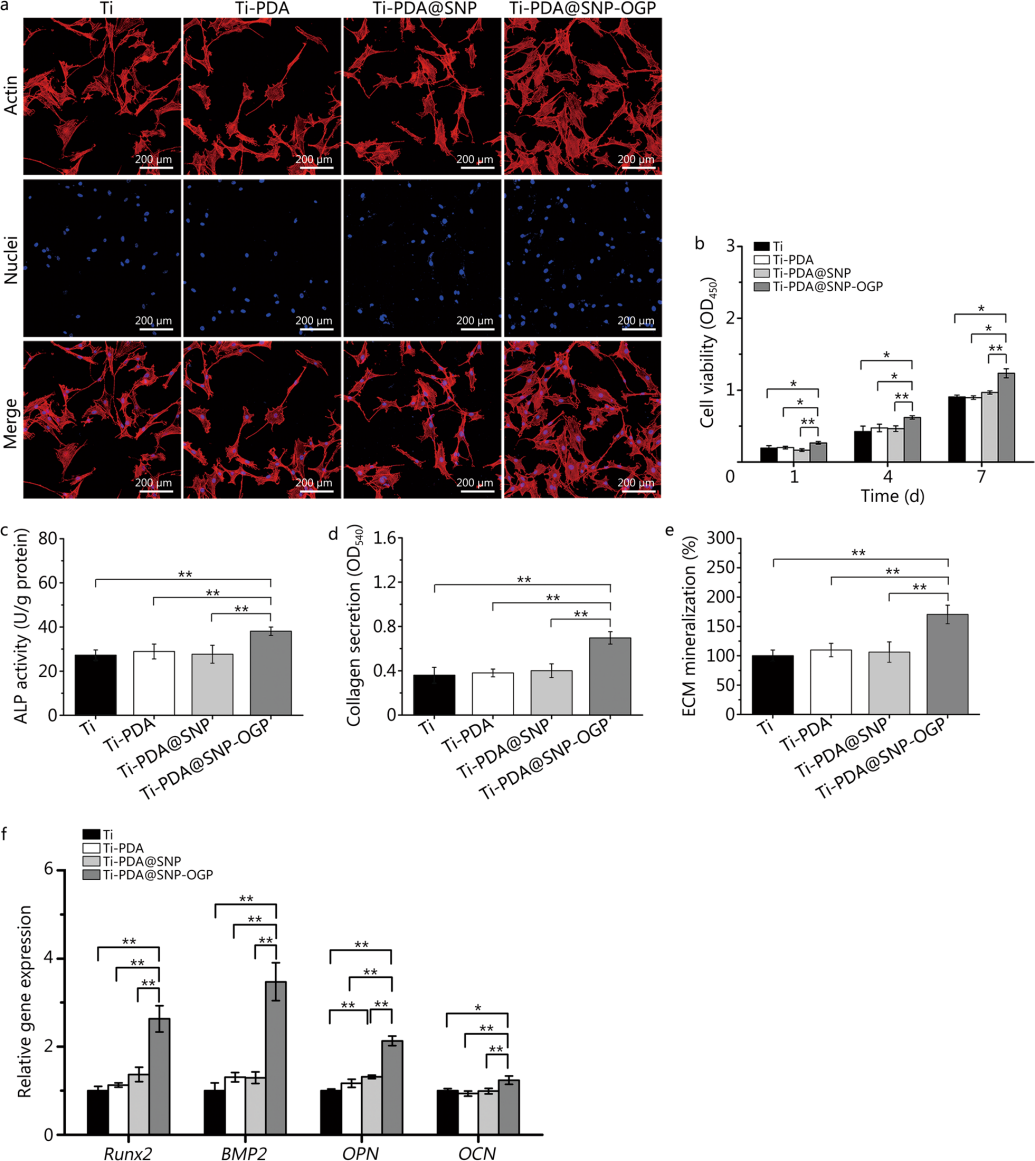
Cell proliferation and osteogenesis evaluation of MSCs on Ti or functionalized Ti substrate. **a** Fluorescence images of MSCs on Ti or functionalized Ti substrate, Hoechst 33258 (blue) and Actin (red). Scale bar = 200 μm. **b** Cell proliferation of MSCs on Ti or functionalized Ti substrate by CCK-8 assay. **c** ALP activity of MSCs on Ti or functionalized Ti substrate after incubation for 7 d. **d** Collagen secretion of MSCs on Ti or functionalized Ti substrate were detected and quantified by Sirius red staining. **e** ECM mineralization of MSCs in each sample were measured and quantified by Alizarin red staining. **f** mRNA expression of osteogenesis-related genes *Runx2*, *BMP2*, *OPN*, and *OCN* measured by qRT‑PCR. ^*^P < 0.05, ^**^P < 0.01; Ti titanium, PDA polydopamine nanoparticles, SNP sodium nitroprusside, OGP osteogenic growth peptide, MSCs marrow stromal cells, ALP alkaline phosphatase, ECM extracellular matrix, Runx2 runt-related transcription factor 2, BMP2 bone morphogenetic protein 2, OPN osteopontin, OCN osteocalcin, qRT‑PCR quantitative real‑time reverse transcription‑polymerase chain reaction

The morphology and spread area of MSCs were evaluated using fluorescent staining. As shown in Fig. 3a, MSCs cultured on Ti or functionalized Ti substrate exhibited spindle-shaped morphology. More pseudopods were identified from MSCs cultured on Ti-PDA@SNP-OGP. Quantitative analysis indicated that the spread area of MSCs was larger on Ti-PDA@SNP-OGP, compared with other groups (*P* < 0.01, Additional file 1: Fig. S4a). Cell viability of MSCs was evaluated using CCK-8 assay after culturing for 1, 4, and 7 d. Compared with cells cultured on Ti, Ti-PDA, and Ti-PDA@SNP, cell viability of MSCs cultured on Ti-PDA@SNP-OGP was significantly higher (*P* < 0.05 or* P* < 0.01, Fig. 3b). After NIR irradiation for 10 min, cell viability of MSCs in Ti was reduced to 85.4%, which was higher than those in Ti-PDA (68.9%), Ti-PDA@SNP (53.2%), and Ti-PDA@SNP-OGP (58.8%), respectively (*P* < 0.01, Additional file 1: Fig. S4b). Meanwhile, cell viabilities in Ti-PDA@SNP and Ti-PDA@SNP-OGP were significantly lower than that in Ti-PDA (*P* < 0.05, Additional file 1: Fig. S4b). Then the cells were further cultured at a normal condition, and it was found that cell viability of MSCs was significantly decreased on Ti-PDA@SNP-OGP than that on Ti after 1 d (*P* < 0.01). No statistically significant differences were identified on day 4 between Ti and Ti-PDA@SNP-OGP groups (*P* > 0.05), whereas a significant difference was found on day 7 (*P* < 0.01, Additional file 1: Fig. S4c). The cell viability (determined using LDH assay) of MSCs cultured on NIR-irradiated Ti-PDA@SNP-OGP approximated that of the positive control group (MSCs cultured on Ti without MSRA). However, after NIR irradiation, cell viabilities in Ti, Ti-PDA, and Ti-PDA@SNP were significantly lower than that in Ti-PDA@SNP-OGP (*P* < 0.01, Additional file 1: Fig. S4d). After the biofilm elimination, we performed the fluorescent staining and ALP activity assay to evaluate the osteogenic potential of Ti-PDA@SNP-OGP. Ti-PDA@SNP-OGP (virgin) without NIR irradiation was used as a control. MSCs cultured on Ti-PDA@SNP-OGP (used) and Ti-PDA@SNP-OGP (virgin) exhibited similar normal morphologies and no obvious differences were found, indirectly reflecting that NIR-irradiated Ti-PDA@SNP-OGP could effectively eradicate MRSA biofilms but not affect the biological function of MSCs (Additional file 1: Fig. S4e). Moreover, the ALP activity of MSCs in Ti-PDA@SNP-OGP (after MRSA biofilms were eradicated) was significantly higher (*P* < 0.05) than that in Ti (Additional file 1: Fig. S4f).

After culturing for 7 d, MSCs that were cultured on Ti-PDA@SNP-OGP exhibited higher ALP activity than those in other groups (*P* < 0.01, Fig. 3c). A similar trend was observed for collagen secretion and ECM mineralization (Fig. 3d, e). qRT‑PCR was used to investigate the mRNA expression profiles of osteogenesis-related genes [Runt-related transcription factor 2 (*Runx2*), bone morphogenetic protein 2 (*BMP2*), osteopontin (*OPN*), osteocalcin (*OCN*)] in MSCs, and the expression levels of *Runx2*, *BMP2*, *OPN* and *OCN* was found to be remarkably higher in Ti-PDA@SNP-OGP than other groups (*P* < 0.05 or *P* < 0.01, Fig. 3f).

### Macrophage phenotype reprogramming and anti-inflammation evaluation in vitro

**Fig. 4:**
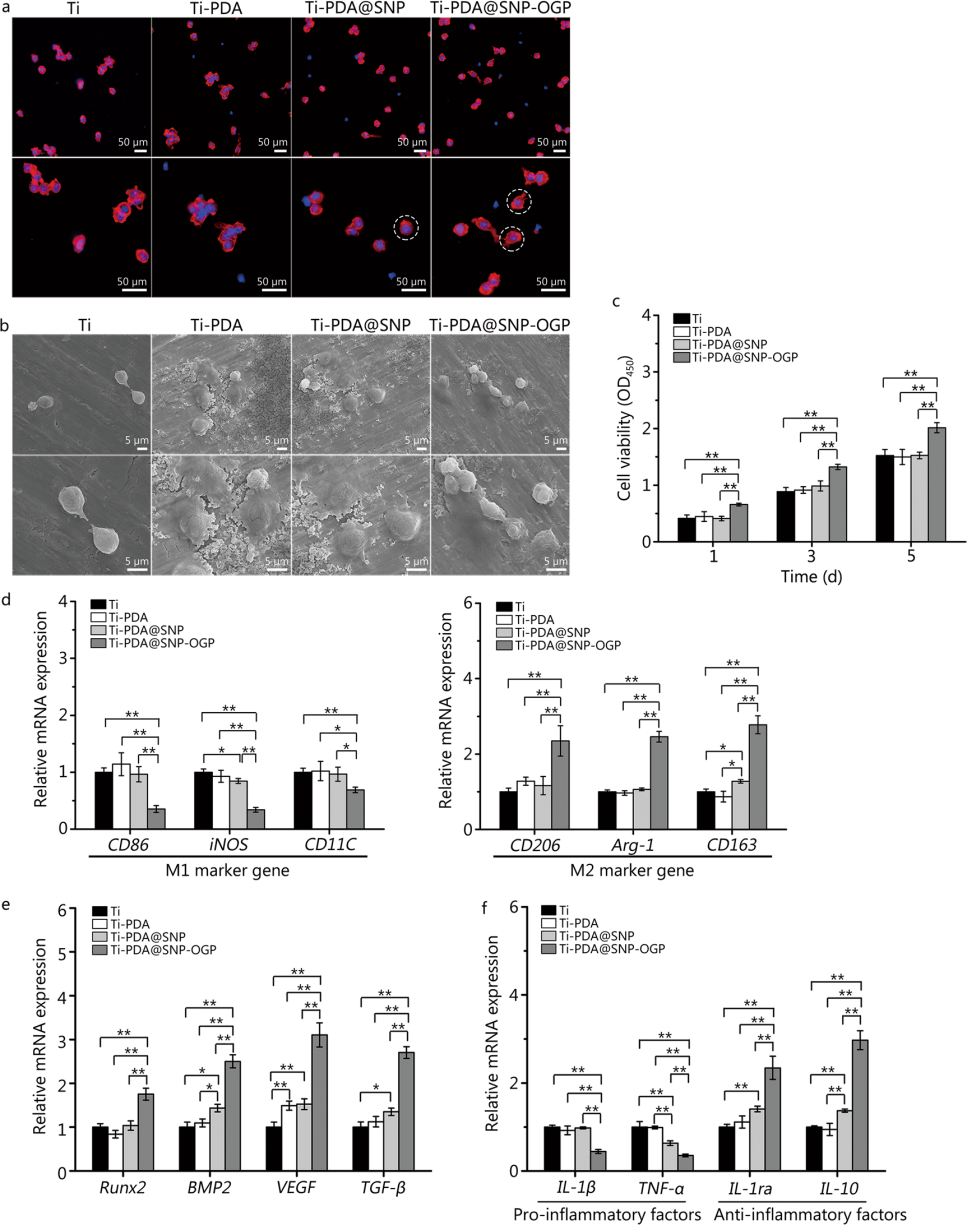
Effects of Ti or functionalized Ti substrate on macrophage phenotype reprogramming and anti-inflammation capacity in vitro. **a** Cytoskeleton staining of RAW 264.7 cells on Ti or functionalized Ti substrate after culturing for 24 h, Hoechst 33258 (blue) and Actin (red). Scale bar = 50 μm. White dotted circles represent the pseudopodium. **b** Scanning electron microscopy (SEM) images of RAW264.7 cells on Ti or functionalized Ti substrate after culturing for 24 h. Scale bar = 5 μm. **c** Cell viability of RAW264.7 cells on various samples after cultured for 1, 3, and 5 d. **d** mRNA expression of M1 marker genes *CD86*, *iNOS* and *CD11C* and M2 marker genes *CD206*, *Arg-1* and *CD163* in lipopolysaccharide (LPS)-stimulated RAW264.7 cells. **e** mRNA expression of *Runx2*, *BMP2*, *VEGF* and *TGF-β* genes in RAW264.7 cells. **f** mRNA expression of pro-inflammatory genes *IL-1β* and *TNF-α* and anti-inflammatory genes *IL-1ra* and *IL-10* in LPS-stimulated RAW264.7 cells. ^*^P < 0.05, ^**^P < 0.01; Ti titanium, PDA polydopamine nanoparticles, SNP sodium nitroprusside, OGP osteogenic growth peptide, CD86 cluster of differentiation 86, iNOS inducible nitric oxide synthase, CD11C cluster of differentiation 11C, CD206 cluster of differentiation 206, Arg-1 arginase-1, CD163 cluster of differentiation 163, Runx2 runt-related transcription factor 2, BMP2 bone morphogenetic protein 2, VEGF vascular endothelial growth factor, TGF-β transforming growth factor-β, IL-1β interleukin-1β, TNF‑α tumor necrosis factor-α, IL-1ra interleukin-1ra, IL-10 interleukin-10

The morphology of RAW264.7 cells cultured on Ti or functionalized Ti substrate was first observed by fluorescent staining. More pseudopodia (indicated by white circles with dotted lines) were observed in RAW264.7 cells cultured on Ti-PDA@SNP-OGP, compared with those cultured on Ti, Ti-PDA or Ti-PDA@SNP (Fig. 4a). SEM images exhibited that, compared with other groups, more RAW264.7 cells adhered to the Ti-PDA@SNP-OGP substrate (Fig. 4b) Moreover, Ti-PDA@SNP-OGP significantly enhanced the proliferation of RAW264.7 cells compared with other groups (*P* < 0.01, Fig. 4c). Moreover, the polarization of the macrophages was also evaluated by qRT-PCR. As shown in Fig. 4d, the M1 marker genes (*CD86*, *iNOS* and *CD11C*) displayed a significant downtrend by Ti-PDA@SNP-OGP compared with other groups (*P* < 0.05 or *P* < 0.01), suggesting its anti-inflammatory activity. By comparison, the mRNA expression levels of M2 marker genes (*CD206*, *Arg-1* and *CD163*) were significantly up-regulated by Ti-PDA@SNP-OGP compared to Ti, Ti-PDA or Ti-PDA@SNP (*P* < 0.01).

The mRNA expression levels of *Runx2*, *BMP2*, *VEGF* and *TGF-β* were further investigated in RAW264.7 cells. Compared with Ti, mRNA expression levels of *Runx2*, *BMP2*, *VEGF* and *TGF-β* in Ti-PDA@SNP-OGP increased up to 1.8, 2.5, 3.1 and 2.7 times, respectively (*P* < 0.01, Fig. 4e). The anti-inflammatory potential of Ti-PDA@SNP-OGP was also evaluated. Compared with other groups, the mRNA expression levels of pro-inflammatory genes *IL-1β* and *TNF-α* were down-regulated in Ti-PDA@SNP-OGP, whereas it showed the opposite tendency with higher mRNA expression of anti-inflammatory genes *IL-1ra* and *IL-10* (*P* < 0.01, Fig. 4f). Collectively, these results highlight the promotion effect of Ti-PDA@SNP-OGP to reverse the adverse pro-inflammatory microenvironment and to reprogram the macrophages into M2 phenotype, therefore creating a pro-regenerative microenvironment.

### In vitro osteo-immunomodulation

The osteogenic differentiation potential of MSCs and expression of inflammatory factors by RAW264.7 cells after NIR irradiation were evaluated. No statistically significant differences were found between Ti-PDA@SNP-OGP and NIR-irradiated Ti-PDA@SNP-OGP (*P* > 0.05), whereas mineralization of MSCs in Ti-PDA@SNP-OGP was higher than other groups after NIR irradiation (*P* < 0.01, Additional file 1: Fig. S4g). Furthermore, after NIR irradiation for 10 min, the mRNA expression level of M1 marker *CD86* in Ti-PDA@SNP-OGP in RAW264.7 cells was significantly lower than other groups (*P* < 0.01, Additional file 1: Fig. S4h). In addition, no statistically significant differences in the expression level of *CD86* between Ti-PDA@SNP-OGP and NIR-irradiated Ti-PDA@SNP-OGP (*P* > 0.05). An opposite trend was observed for M2 marker *CD206* expression in RAW264.7 cells (Additional file 1: Fig. S4h). After biofilm elimination, the interaction of MSCs with the used Ti-PDA@SNP-OGP was investigated in vitro using CCK-8 and ALP activity assays. MSCs on Ti-PDA@SNP-OGP used for the first or second time exhibited significantly higher cell viability than those on Ti or Ti-PDA@SNP-OGP used for three time (*P* < 0.05 or *P* < 0.01, Additional file 1: Fig. S4i). Similarly, the ALP activities of MSCs on Ti-PDA@SNP-OGP used for the first or second time were higher than those on Ti or Ti-PDA@SNP-OGP used three time after incubation for 7 d (*P* < 0.05, Additional file 1: Fig. S4j). Collectively, the results indicate that hyperthermia and photothermally-triggered NO release by Ti-PDA@SNP-OGP can be repeated two times to eradicate the established biofilms and to improve MSCs osteogenic differentiation after biofilm eradication.

RAW264.7 cells exposed to Ti-PDA@SNP-OGP were found to have significantly enhanced mRNA and protein expressions of *Runx2*, *BMP2*, *VEGF* and *TGF-β* (*P* < 0.01, Additional file 1: Fig. S5a, b). Furthermore, a Transwell co-culture system was utilized to further evaluate the in vitro promotion osteogenic effect of RAW264.7 cells induced by Ti-PDA@SNP-OGP on MSCs. RAW264.7 cells were seeded on different substrates in the bottom chamber, and MSCs were cultured in the upper chamber (Additional file 1: Fig. S5c). After co-culture for 1 d, more migrated MSCs were found in Ti-PDA@SNP-OGP (Additional file 1: Fig. S5d), which had the highest transmembrane migration among all groups (*P* < 0.05 or* P* < 0.01). Quantitative analysis showed that ALP activity, the levels of collagen secretion and ECM mineralization of MSCs were significantly higher in Ti-PDA@SNP-OGP than in other groups (*P* < 0.01). Similarly, larger Sirius red-stained areas of collagen fibers and Alizarin red-stained areas of calcium deposits were found in Ti-PDA@SNP-OGP (Additional file 1: Fig. S5e). Furthermore, the expression levels of osteogenesis-related genes *Runx2*, *BMP2*, *ALP*, *OPN*, and *OCN* were the highest in Ti-PDA@SNP-OGP group (*P* < 0.01, Additional file 1: Fig. S5f).

### Microbiological evaluation in vivo

To verify the in vitro results, an MRSA-infected femoral defect implantation model was successfully established (Additional file 1: Fig. S6a). SEM images showed that Ti-PDA@SNP-OGP was not damaged during implantation (Additional file 1: Fig. S6b). Before implantation, Ti and Ti-PDA@SNP-OGP rods were incubated with MRSA biofilms for 2 d. Formation of MRSA biofilms on implants was examined with SEM. It was found that all implants were covered with MRSA biofilms after incubation with MRSA suspension for 2 d (Additional file 1: Fig. S6c). The adherent MRSA on all implants was exfoliated by ultrasonic energy. Quantitative analysis indicated that MRSA could be found in Ti (2.71 × 10^7^ CFU), Ti-PDA (2.60 × 10^7^ CFU), Ti-PDA@SNP (2.46 × 10^7^ CFU), and Ti-PDA@SNP-OGP (2.66 × 10^7^ CFU).

One day after the implantation, the implantation sites of Ti or Ti-PDA@SNP-OGP implant were exposed to laser irradiation at 808 nm. Photothermal images and corresponding temperature changes were recorded (Additional file 1: Fig. S6d). After irradiation for 10 min, the temperature change (∆T) of the Ti-PDA@SNP-OGP implant was 23.7 °C, which was significantly higher than that of the Ti implant (12.1 °C,* P* < 0.01, Additional file 1: Fig. S6e). After implantation for 3 d, both implants were gently removed and soaked into MHB for 12 h. After incubation in the dark, the MHB medium containing the Ti or Ti-PDA@SNP-OGP implant was feculent. This is indicative of the insufficient antibacterial capabilities of Ti and Ti-PDA@SNP-OGP without NIR irradiation. After NIR irradiation, the MHB medium containing Ti-PDA@SNP-OGP implant became clear, while the MHB medium containing the Ti implant remained feculent (Additional file 1: Fig. S6f). Quantitative analysis of the spread plates showed that only 6.2% of MRSA biofilms were eliminated without NIR irradiation. In contrast, over 95.7% of MRSA biofilms on Ti-PDA@SNP-OGP implant were eradicated after NIR irradiation (*P* < 0.01, Additional file 1: Fig. S6g). Inductively coupled plasma-atomic emission spectroscopy (ICP-AES) was used to investigate the content of Fe ions in the intraluminal femoral marrow to determine the in vivo degradation of Ti-PDA@SNP-OGP at pre-determined time-points (0.5, 1, 3, 7, 14 and 28 d). The percentage of Fe ions that accumulated in the intraluminal femoral marrow was 7.54% in Ti-PDA@SNP-OGP at 28 d. Notably, the half-life of OGP in Ti-PDA@SNP-OGP substrate in vivo was approximately 7 d. In contrast, the cumulative amount of Fe ions in Ti was only 0.9% (*P* < 0.01, Additional file 1: Fig. S6h). Collectively, the microbiological evaluation demonstrated the Ti-PDA@SNP-OGP implant displayed a combinational photothermal and NO antibacterial and MRSA biofilm eradication effect in vivo.

### Anti-inflammation and macrophage phenotype reprogramming in vivo

To evaluate the anti-inflammatory potential, ELISA was carried out to quantify the concentration of excretive pro-inflammatory (TNF-α and IL-6) and anti-inflammatory cytokines (TGF-β and IL-10). NIR-irradiated Ti-PDA@SNP-OGP had the lowest protein expression levels of TNF-α and IL-6 (*P* < 0.01, Additional file 1: Fig. S6i). The contents of TNF-α and IL-6 were significantly higher in Ti-PDA@SNP-OGP than those in NIR-irradiated Ti-PDA@SNP-OGP, whereas it showed an opposite tendency with higher contents of anti-inflammatory cytokines TGF-β and IL-10 secreted by the latter (*P* < 0.01, Additional file 1: Fig. S6i), which was ascribed to photothermally-triggered NO generation.

HE and Giemsa stainings were used to analyze the inflammatory response 3 d after implantation in vivo. Only a few inflammatory cells (indicated by red arrows) and residual bacteria (indicated by red arrows) were identified in NIR-irradiated Ti-PDA@SNP-OGP, which was further confirmed by quantitative results (*P* < 0.05 or* P* < 0.01, Additional file 1: Fig. S7a). Conversely, profuse infiltration of inflammatory cells and many MRSA cells were observed in other groups (*P* < 0.01, Additional file 1: Fig. S7b).

At the bone-implant interface, IHC staining for M1 macrophages with CD86 and M2 macrophages with CD206 was performed (Additional file 1: Fig. S7c). In NIR-irradiated Ti-PDA@SNP-OGP, the distribution of CD86 positive macrophages presented a remarkable decrease, whereas they displayed a predominantly stronger trend of CD206 positive macrophages. Collectively, these results demonstrate the potential of the implant to mediate anti-inflammation and macrophage phenotype reprogramming, in line with the in vitro results.

### Bone regeneration and biosafety evaluation in vivo

**Fig. 5:**
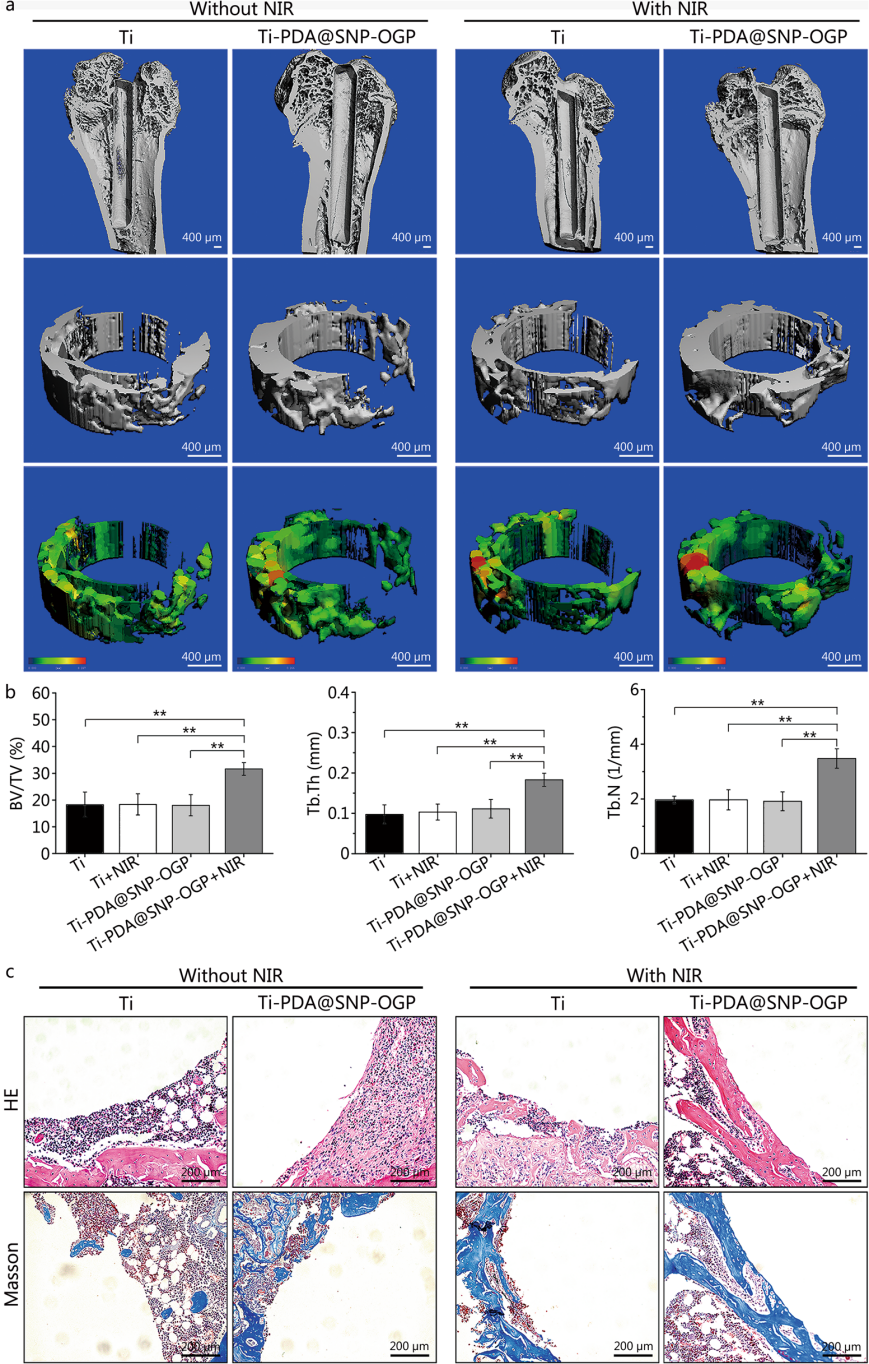
Bone regeneration in an MRSA-infected femoral defect implantation model in vivo. **a** Micro-CT images of new bone. Scale bar = 400 μm. **b** Quantitative analysis of newly-formed bone tissues based on three‑dimensional (3D) reconstruction images of micro-CT, including bone volume/total volume (BV/TV), trabecular thickness (Tb.Th), and trabecular number (Tb.N) after 4 weeks. **c** Representative images of HE and Masson’s trichrome staining at bone-implant interface. Scale bar = 200 μm. ^**^P < 0.01; Ti titanium, PDA polydopamine nanoparticles, SNP sodium nitroprusside, OGP osteogenic growth peptide, HE hematoxylin and eosin

As shown in Fig. 5a, more new bone formation was observed in Ti-PDA@SNP-OGP group that had been subjected to NIR irradiation. The percentages of new bone volume over total bone volume (BV/TV), the trabecular plate thickness (Tb.Th), trabecular number (Tb.N) and trabecular separation (Tb.Sp) were investigated and calculated. The highest percentages of BV/TV, Tb.Th, and Tb.N and the lowest percentage of Tb.Sp were found in NIR-irradiated Ti-PDA@SNP-OGP (Fig. 5b, Additional file 1: Fig. S7d). As shown in Fig. 5c, abundant new bone tissue formation was observed on the surface of NIR-irradiated Ti-PDA@SNP-OGP, while only a small amount of new bone was found in Ti, NIR-irradiated Ti or Ti-PDA@SNP-OGP after 4 weeks of treatment. Similar results were obtained for Masson’s trichrome staining. NIR-irradiated Ti-PDA@SNP-OGP exhibited the highest percentage of the new bone area (37.5%) and bone-to-implant contact (34.3%) (*P* < 0.05 or* P* < 0.01, Additional file 1: Fig. S7e), consistent with the results of micro-CT. Further evaluation of the bone-forming related proteins (ALP and OPN) around the peri-implant bone tissues with IHC staining identified more positively-stained areas of ALP and OPN in Ti-PDA@SNP-OGP. Compared with Ti, NIR-irradiated Ti, and Ti-PDA@SNP-OGP, NIR-irradiated Ti-PDA@SNP-OGP showed significantly improved CD31 expression (vascularization marker) around the implants (Additional file 1: Fig. S7f).

Biosafety of the mild temperature (~51 °C) induced by PTT of Ti-PDA@SNP-OGP in vivo was evaluated using whole blood biochemical analysis, including white blood cells (WBC), neutrophil granulocytes (NEUT), red blood cells (RBC), hemoglobin (HGB), hematocrit (HCT), and platelets (PLT). No statistically significant differences were found between Ti and NIR-irradiated Ti-PDA@SNP-OGP for the WBC, NEUT, RBC, HGB, HCT, and PLT indicators (Additional file 1: Fig. S8). These results indicate that there were no obvious risks associated with Ti-PDA@SNP-OGP and NIR therapy inside the body.

## Discussion

**Fig. 6:**
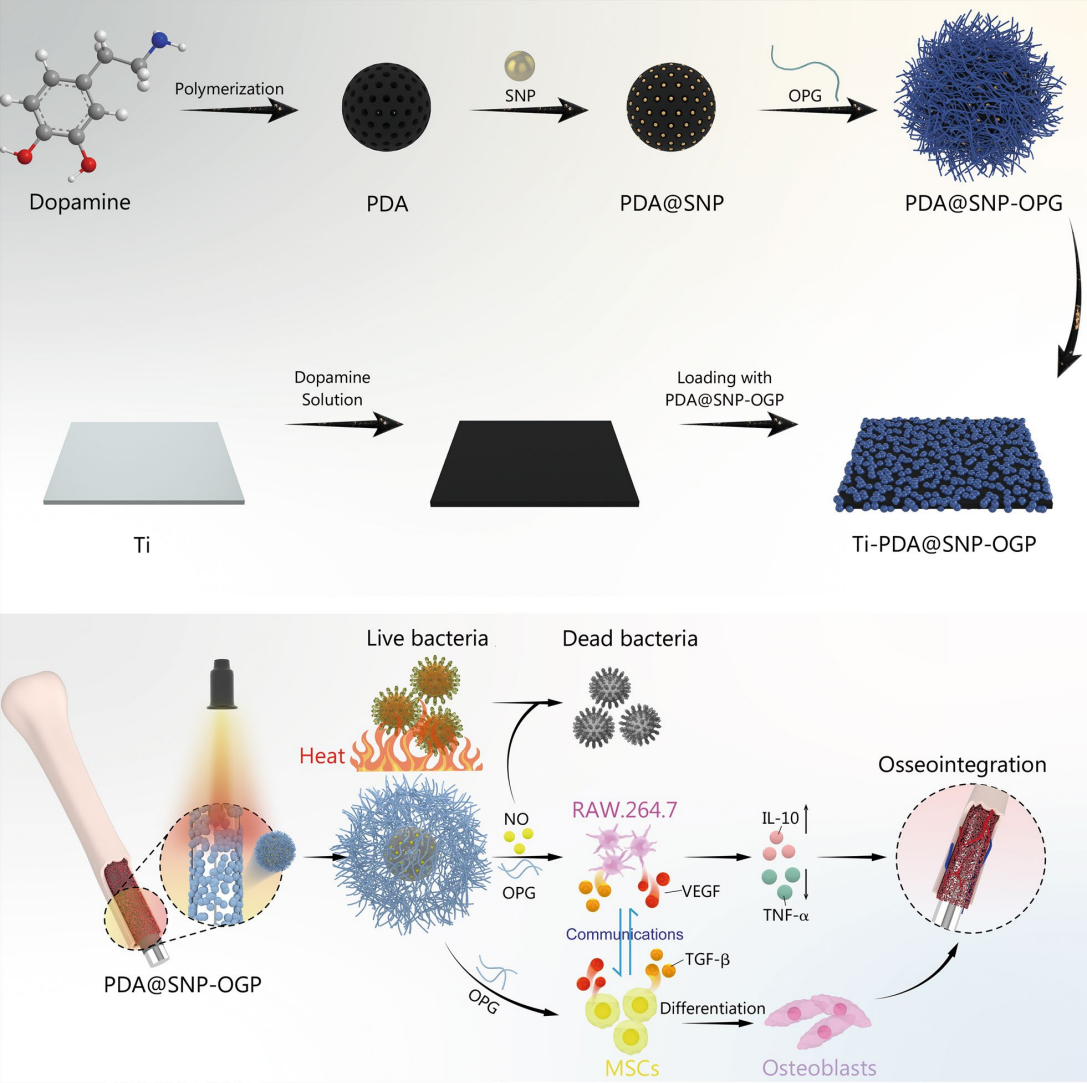
Schematic diagrams showing that Ti-PDA@SNP-OGP eliminates MSRA biofilms via photothermally-triggered NO and immunotherapy for enhanced osseointegration. Ti titanium, PDA polydopamine nanoparticles, SNP sodium nitroprusside, OGP osteogenic growth peptide, NO nitric oxide, VEGF vascular endothelial growth factor, TGF-β transforming growth factor-β, IL-10 interleukin-10, TNF‑α tumor necrosis factor-α

In this study, PDA nanoparticles were encapsulated with photothermally-sensitive SNP and modified using OGP, and a dopamine coating was formed on Ti to load PDA@SNP-OGP nanoparticles and construct Ti-PDA@SNP-OGP (Fig. 6). As an osteogenic factor, OGP has been widely used to augment the osteogenic differentiation potential of bone-repairing materials by improving the proliferation and differentiation of osteoblast lineage cells in vivo*.* In our previous studies, OGP was identified to be an effective osteo-immunomodulatory factor for improving the anti-inflammation capability of RAW264.7 cells and augmenting the osteogenic effect of MSCs [41–43]. Bai et al. [44] fabricated a tetravalent catechol-containing (DOPA)_4_-modified OGP with mussel adhesion and osteo-immunomodulatory functions for advanced osseointegration via suppression of inflammatory responses and up-regulating the M2 phenotype of macrophages. However, the poor antibacterial capacity rendered the OGP-modified Ti implants extremely limited applications in clinic [44–46]. Numerous studies have focused only on the functionalization of OGP-modified Ti implants, such as single function (antibacterial capacity or enhanced osseointegration) or dual function (including antibacterial capacity and enhanced osseointegration). However, those approaches do not improve implant osseointegration in the presence of multiple co-morbidities (e.g., bacterial infection, inflammation). The emergence of MRSA and its biofilms on the surfaces of Ti implants exacerbates the situation, therefore resulting in the failure of Ti implants [47]. For this reason, we designed a multi-functionalized coating on Ti that could regulate the crosstalk between RAW264.7 cells and MSCs to enhance osseointegration and provide antibacterial and biofilm-eliminating capacities using NIR irradiation.

Due to the excellent photothermal effect of PDA, the localized hyperthermia induced by laser-irradiated light energy for the elimination of established biofilms was widely employed [21, 23]. The temperature increment by a photothermal agent is related to factors such as its concentration, laser density, laser irradiation time and ambient temperature [20, 21]. However, the biofilms are only eradicated by the application of NIR irradiation at 70 °C. Such a temperature unavoidably reduces host cell viability by inducing apoptosis or necrosis [48]. In contrast, the mild temperature (~52 °C) induced by PTT results in fewer adverse effects, but the antibacterial and biofilm-eradicating capabilities are also significantly reduced at that mild temperature [23]. In this study, Ti-PDA@SNP-OGP converted laser-irradiated light energy into localized hyperthermia. The increased tissue temperature, in turn, triggered NO release from SNP in an “on-demand” manner due to the destruction of π-π stacking and/or physical adsorption between SNP and PDA nanoparticles [40]. To minimize the adverse effects, 0.5 mg/ml of PDA@SNP-OGP nanoparticles with the laser intensity of 1.0 W/cm^2^ for 10 min was employed to investigate the photothermal effect and the temperature reached about to 52 °C. The hyperthermia gathered by Ti-PDA@SNP-OGP could destroy the bacterial membrane and subsequently cause the protein leakage of MRSA, resulting in the desirable antibacterial capacity. These results are consistent with previous studies [49, 50]. The release of NO can improve the antibacterial effect by causing nitrosative and oxidative stresses and breaking the bacterial nitrogen metabolism [51, 52]. Based on these results, the controllable and rapid release of NO via NIR irradiation is a useful armamentarium for the elimination of biofilms within a short time and does not require exorbitant temperatures.

Ideal implants should present excellent cytocompatibility for regulating the fundamental functions of osteogenesis-related cells [53, 54]. Cell viability, ALP activity, collagen secretion, and ECM mineralization are significantly improved by Ti-PDA@SNP-OGP. In this study, the osteogenesis-related genes *Runx2*, *BMP2*, *OPN*, and *OCN* were significantly up-regulated in Ti-PDA@SNP-OGP compared with other groups. These results indicate that Ti-PDA@SNP-OGP has the potential to improve the osteogenic differentiation of MSCs [42, 43].

Once implant has been imbedded into bone tissues, immune cells such as macrophages are recruited to the implant site within a few hours; these immune cells generate a cascade of events that lead to the early inflammatory response [55–59]. An inflammatory response is beneficial for improving bone healing because bone MSCs are recruited and angiogenesis is stimulated [60–62]. In the late stage of inflammation, M1 macrophages will polarize into the M2 phenotype to alleviate inflammation and secrete anti-inflammatory cytokines to facilitate bone regeneration [63–67]. Nevertheless, the multi-functional macrophages are not able to undergo phenotypic transition under pathological conditions [59]. Persistent and severe inflammation results in elevated levels of pro-inflammatory cytokines in the surrounding bone tissues. These pro-inflammatory cytokines augment the activity of osteoclasts and decrease the recruitment or migration of MSCs. This results in bone disconnection and poor osseointegration [68–72]. In this study, Ti-PDA@SNP-OGP significantly down-regulated the mRNA expression levels of M1 markers CD86, iNOS, and CD11C and up-regulated the mRNA expression levels of M2 markers CD206, Arg-1, and CD163. Besides, the mRNA expression levels of *Runx2*, *BMP2*, *VEGF*, and *TGF-β* genes were significantly up-regulated by Ti-PDA@SNP-OGP. Additionally, compared with other groups, Ti-PDA@SNP-OGP also inhibited the mRNA expression levels of pro-inflammatory cytokines IL-1β and TNF-α and enhanced the expression of anti-inflammatory cytokines IL-1ra and IL-10. This could be due to the osteo-immunomodulatory effect of Ti-PDA@SNP-OGP [42, 46].

The crosstalk between MSCs and RAW264.7 cells plays a vital role in the improvement of implant osseointegration [72–74]. Therefore, the osteogenic induction effect of RAW264.7 cells on MSCs upon the stimulation of Ti-PDA@SNP-OGP should be carefully considered. The mRNA and protein expressions of *Runx2, BMP2, VEGF,* and *TGF-β* in RAW264.7 cells were up-regulated, which might be attributed to the immobilization of OGP on Ti to stimulate RAW264.7 cells to secrete osteogenic mediators [44, 46]. The MSCs in Ti-PDA@SNP-OGP exhibited the highest transmembrane migration among all groups after co-culture with RAW264.7 cells. Furthermore, ALP activity, collagen secretion and ECM mineralization in Ti-PDA@SNP-OGP were higher than those in Ti, Ti-PDA or Ti-PDA@SNP. Additionally, Ti-PDA@SNP-OGP was proven to stimulate the expression of osteogenesis-related genes *Runx2*, *BMP2*, *ALP*, *OPN* and *OCN* in MSCs. Based on the above results, we suggested that Ti-PDA@SNP-OGP is a potentially effective bone immuno-modulator that enhances the release of anti-inflammatory mediators from RAW264.7 cells to improve the migration and differentiation of MSCs via multiple paracrine signalings of Runx2, BMP2, VEGF, and TGF-β.

For the evaluation of antibacterial and anti-inflammatory abilities in vivo, over 95.7% of MRSA biofilms on Ti-PDA@SNP-OGP implants were eradicated after NIR irradiation. Besides, only a few inflammatory cells and residual bacteria were observed in Ti-PDA@SNP-OGP after NIR irradiation, due to the synergistic effects of mild hyperthermia and photothermally-triggered NO release on MRSA biofilm elimination [17, 23]. The potential antibacterial mechanisms for our designed Ti-PDA@SNP-OGP were elaborated below. Firstly, hyperthermia makes bacteria more sensitive to the external environment and can subsequently efficiently damage the bacterial membrane, which may play a dominant role in the process of MRSA-biofilm elimination. Secondly, photothermally-triggered NO could break the nitrogen metabolism, induce nitrosative/oxidative stresses, and lead to MRSA death. Thirdly, immunotherapy can improve the host’s defense against invasive bacteria and enhance the antibacterial property against the residual bacteria [75]. Besides, Ti-PDA@SNP-OGP possesses a better ability to suppress inflammatory responses via down-regulation of pro-inflammatory cytokines TNF-α and IL-6 and up-regulation of anti-inflammatory cytokines TGF-β and IL-10. The enhanced anti-inflammatory response might be attributed to the excellent antibacterial activity of NIR-irradiated Ti-PDA@SNP-OGP and the immuno-modulatory effect of OGP [42]. The in vivo ICP-AES results suggested that Ti-PDA@SNP-OGP was mainly distributed in the bone marrow cavity of the femurs. The Ti-PDA@SNP-OGP coating was quickly degraded within the first 7 d, followed by a declined degradation over the next 21 d. These results demonstrated that Ti-PDA@SNP-OGP possessed sustained degradation characteristics. Such a feature is beneficial for enhanced osseointegration.

Micro-CT confirmed that the highest percentages of BV/TV, Tb.Th, and Tb.N and the lowest percentage of Tb.Sp were found in Ti-PDA@SNP-OGP. The IHC staining of bone-forming related proteins ALP, OPN, and CD31 suggested that NIR-irradiated Ti-PDA@SNP-OGP presented better osteogenesis compared with other groups, as shown by the higher expression of ALP, OPN and OCN. Collectively, excellent osseointegration was simultaneously achieved due to the osteo-immunomodulatory effect of Ti-PDA@SNP-OGP.

Several limitations need to be further addressed. The photothermal effect on the physiological functions of MSCs and RAW264.7 cells should be explored further. Whether the bioactive properties of OGP after NIR irradiation could be inactivated and the in vivo release of NO in an infected femur implantation model should be thoroughly investigated. NO is an inflammatory molecule, thus the feedback of NO to macrophages and its potential adverse influences on the surrounding tissues should be further investigated. In terms of cell behaviors, it exhibits dynamic, coupled, and spatiotemporally regulated properties, yet the physiological functions of MSCs, and macrophages were not controlled precisely by our fabricated Ti implants in response to spatiotemporal cues. Therefore, the fabrication of multi-functional Ti implants that achieves precise control of the physiological functions of MSCs and RAW264.7 cells as well as excellent antibacterial and biofilm-eradicating properties could be a more rational approach for enhanced osseointegration.

## Conclusions

In this study, we for the first time proposed a novel NIR-activatable multi-functional interface with responsive NO-potentiated mild PTT and osteo-immunomodulatory OGP on Ti implants, based on a PDA-mediated interfacial functionalization, for eradication of MRSA biofilms and enhanced osseointegration. We demonstrated that Ti-PDA@SNP-OGP is an ideal platform for controlled NO release. Specifically, NO was loaded on Ti-PDA@SNP-OGP through a special π-π stacking interaction and/or physical adsorption between the SNP molecule and PDA. Ti-PDA@SNP-OGP was found to display a strong photothermal effect upon 808 nm laser irradiation, which provided an ideal external stimulating condition for the controlled release of NO. In particular, NO release could be precisely controlled by intermittent NIR irradiation, showing an “on–off” switch mode.

Upon NIR irradiation, Ti-PDA@SNP-OGP displayed a synergistic photothermal and NO antibacterial effect by significantly inhibiting the growth of MRSA. Moreover, the biofilms formed by MRSA were effectively eliminated by the combinational photothermal and NO treatment. The antibacterial mechanism indicated that bacteria treated with Ti-PDA@SNP-OGP were sterilized by ROS-mediated oxidative stress, destruction of bacterial membrane integrity and leakage of bacterial contents. In vitro experiments revealed that Ti-PDA@SNP-OGP not only facilitated osteogenic differentiation of MSCs, but also suppressed M1 macrophages, whereas, stimulated pro-healing M2 phenotype, thereby remodeling the damaged microenvironment into a pro-regenerative microenvironment, which, in turn, facilitated osteogenesis and suppressed inflammation via the crosstalk of multi-signaling pathways.

Furthermore, in a rat model of implant-associated infection, Ti-PDA@SNP-OGP eliminated the formed MRSA biofilms, alleviated the accompanying inflammation and mediated the osteo-immunomodulation, resulting in excellent osseointegration. It is noted that both in vitro and in vivo biocompatibility evaluations suggested that Ti-PDA@SNP-OGP and NIR therapy were safe for biomedical applications. Taken together, the study provides a promising strategy for fabricating multi-functional Ti implants to eradicate MRSA biofilms and enhance osseointegration.

## Supplementary Information

**Additional file 1: Table S1.** qRT-PCR primers for MSCs and RAW 264.7 cells used in this study. **Fig. S1.** Characterization of Ti-PDA@SNP-OGP. **Fig. S2.** Characterization of Ti-PDA@SNP-OGP substrate. **Fig. S3.** Mechanism of inhibition on methicillin-resistant Staphylococcus aureusbiofilms. **Fig. S4.** Biocompatibility, anti-inflammation, and duplication evaluation of Ti or functionalized Ti substrate. **Fig. S5.** In vitro osteo-immunomodulation of Ti-PDA@SNP-OGP. **Fig. S6.** Antibacterial and anti-inflammatory evaluation in vivo. **Fig. S7.** Anti-inflammation, antibacterial activity and bone regeneration in vivo. **Fig. S8.** Biosafety of the mild temperatureinduced by PTT of Ti-PDA@SNP-OGP in vivo.

## Data Availability

The data and materials used in the current study are all available from the corresponding author upon reasonable request.

## Abbreviations

ALP: Alkaline phosphatase
Arg-1: Arginase-1
ATP: Adenosine triphosphate
BAI: Biomaterials-associated infection
BCA: Bicinchoninic acid
BMP2: Bone morphogenetic protein 2
BNN6: N,N′-disecbutyl-N,N′-dinitroso-p-phenylenediamine
BV/TV: Bone volume over total bone volume
CCK-8: Cell counting kit-8
CFU: Colony forming unit
DAB: 3,3ʹ-Diaminobenzidine
DCF-DA: 2′,7′-Dichlorofluorescein diacetate
DMEM: Dulbecco’s Modified Eagle’s Medium
ECM: Extracellular matrix
ELISA: Enzyme-linked immunosorbent assay
EPS: Extracellular polymeric substances
FTIR: Fourier Transform infrared spectroscopy
GSNO: S-nitrosoglutathione
HCT: Hematocrit
HGB: Hemoglobin
HE: Hematoxylin and eosin
ICP-AES: Inductively coupled plasma atomic emission spectroscopy
IHC: Immunohistochemistry
IL‑6: Interleukin‑6
IL-10: Interleukin‑10
IL‑1β: Interleukin‑1β
Lc1: Loads of cohesion
Lc2: Loads of adhesion
LPS: Lipopolysaccharide
L-Arg: L-arginine
MHB: Mueller hinton broth
MRSA: Methicillin-resistant *Staphylococcus aureus*
MSCs: Marrow stromal cells
NEUT: Neutrophil granulocytes
NIR: Near-infrared light
NO: Nitric oxide
NONOates: Diazeniumdiolates
OCN: Osteocalcin
OGP: Osteogenic growth peptide
ONPG: O-nitrophenyl-β-d-galactopyranoside
OPN: Osteopontin
PDA: Polydopamine nanoparticles
PLT: Platelets
PTT: Photothermal therapy
qRT‑PCR: Quantitative real‑time reverse transcription‑polymerase chain reaction
RBC: Red blood cells
ROS: Reactive oxygen species
Runx2: Runt-related transcription factor 2
SEM: Scanning electron microscopy
SNP: Sodium nitroprusside
Tb.N: Trabecular number
Tb.Sp: Trabecular separation
Tb.Th: Trabecular plate thickness
TGF-β: Transforming growth factor-β
Ti: Titanium
TNF‑α: Tumor necrosis factor-α
VEGF: Vascular endothelial growth factor
WBC: White blood cells
WCA: Water contact angles
XPS: X-ray photo-electron spectroscope
