# Supplementary material for: Elimination of methicillin-resistant Staphylococcus aureus biofilms on titanium implants via photothermally-triggered nitric oxide and immunotherapy for enhanced osseointegration
Source: Mil Med Res. 2023 May 4;10:21. doi: 10.1186/s40779-023-00454-y (PMC10158155; doi:10.1186/s40779-023-00454-y)
Supplement: Supplementary file 1 — Additional file 1: Table S1. qRT-PCR primers for MSCs and RAW 264.7 cells used in this study. Fig. S1. Characterization of Ti-PDA@SNP-OGP. Fig. S2. Characterization of Ti-PDA@SNP-OGP substrate. Fig. S3. Mechanism of inhibition on methicillin-resistant Staphylococcus aureusbiofilms. Fig. S4. Biocompatibility, anti-inflammation, and duplication evaluation of Ti or functionalized Ti substrate. Fig. S5. In vitro osteo-immunomodulation of Ti-PDA@SNP-OGP. Fig. S6. Antibacterial and anti-inflammatory evaluation in vivo. Fig. S7. Anti-inflammation, antibacterial activity and bone regeneration in vivo. Fig. S8. Biosafety of the mild temperatureinduced by PTT of Ti-PDA@SNP-OGP in vivo. [file 40779_2023_454_MOESM1_ESM.pdf]

Table S1 qRT-PCR primers for MSCs and RAW 264.7 cells used in this study

Target gene	Primers sequences
**MSCs**	
Runx2	F: 5’-GCCGTAGAGAGCAGGGAAGAC-3’<br>R: 5’-CTGGCTTGGATTAGGGAGTCAC-3’
BMP2	F: 5’-ACCAGCCTTCCACCCAAA-3’<br>R: 5’-CGCACCCTATCCCTTCACA-3’
OPN	F: 5’-TTGGGATGGAGGGAGTTTA-3’<br>R: 5’-TACAGCACGCTTGTGGATG-3’
OCN	F: 5’-ACTGTCAGAGCTACTACGCA-3’<br>R: 5’-CGGCATCTAACTCTCCGTAG-3’
VEGF	F: 5’-AGCCCATGAAGTGGTGAAGT-3’<br>R: 5’-GCTCACAGTGATTTTCTGGCTT-3’
TGF-β	F: 5’-CCTGGAAAGGGCTCAACAC-3’<br>R: 5’-CAGTTCTTCTCTGTGGAGCTGA-3’
ALP	F: 5’-AGCGACACGGACAAGAAGC-3’<br>R: 5’-GGCAAAGACCGCCACATC-3’
GAPDH	F: 5’-TGTCCCTAATGACAGCTCCTT-3’<br>R: 5’-GCATCCACCCAAATGACACA-3’
**RAW 264.7 cells**	
CD86	F: 5’-CTGCTCATCATTGTATGTCAC-3’<br>R: 5’-ACTGCCTTCACTCTGCATTTG-3’
iNOS	F: 5’-CACCAAGCTGAACTTGAGCG-3’<br>R: 5’-CGTGGCTTTGGGCTCCTC-3’
CD11C	F: 5’-ACTTCACGGCCTCTCTTCC-3’<br>R: 5’-CACCAGGGTCTTCAAGTCTG-3’
CD206	F: 5’-AGACGAAATCCCTGCTACTG-3’<br>R: 5’-CACCCATTCGAAGGCATTC-3’
Arg-1	F:5’-GGAATCTGCATGGGCAACCTGTGT-3’<br>R: 5’-AGGGTCTACGTCTCGCAAGCCA-3’

CD163	F: 5’-CGTGTGCAGTGTCCAAAAGG-3’<br>R: 5’-CACAAACCAAGAGTGCCGTG-3’
Runx2	F: 5’-AAATGCCTCCGCTGTTATGAA-3’<br>R: 5’-GCTCCGGCCCACAAATCT-3’
BMP2	F: 5’-GCTCCACAAACGAGAAAAGC-3’<br>R: 5’-AGCAAGGGGAAAAGGACACT-3’
VEGF	F: 5’-GTCCCATGAAGTGATCAAGTTC-3’<br>R: 5’-TCTGCATGGTGATGTTGCTCTCTG-3’
TGF-β	F: 5’-CAGTACAGCAAGGTCCTTGC-3’<br>R: 5’-ACGTAGTAGACGATGGGCAG-3’
IL-1β	F: 5’-TGCCACCTTTTGACAGTGATG-3’<br>R: 5’-TGATGTGCTGCTGCGAGATT-3’
TNF-α	F: 5’-CAGGCGGTGCCTATGTCTC-3’<br>R: 5’-CGATCACCCCGAAGTTCAGTAG-3’
IL-10	F: 5’-TTGAACCACCCGGCATCTAC-3’<br>R: 5’-CCAAGGAGTTGCTCCCGTTA-3’
IL-1ra	F: 5’-GGGACCTTACAGTCACCTAATCT-3’<br>R: 5’-CTTGCATCTTGCAGGGTCTTT-3’
GAPDH	F: 5’-TGACCACAGTCCATGCCATC-3’<br>R: 5’-GACGGACACATTGGGGGTAG-3’

Runx2 Runt-related transcription factor-2, *BMP2* bone morphogenetic protein-2, *OPN* osteopontin, *OCN* osteocalcin, *VEGF* vascular endothelial growth factor, *TGF-β* transforming growth factor-β, *ALP* alkaline phosphatase, *GAPDH* glyceraldehyde-3-phosphate dehydrogenase, *CD86* cluster of differentiation 86, *iNOS* inducible nitric oxide synthase, *CD11C* cluster of differentiation 11C, *CD206* cluster of differentiation 206, *Arg-1* arginase-1, *CD163* cluster of differentiation 163, *IL-1β* interleukin-1β, *TNF-α* tumor necrosis factor-α, *IL-10* interleukin-10, *IL-1ra* interleukin-1ra, *GAPDH* glyceraldehyde-3-phosphate dehydrogenase

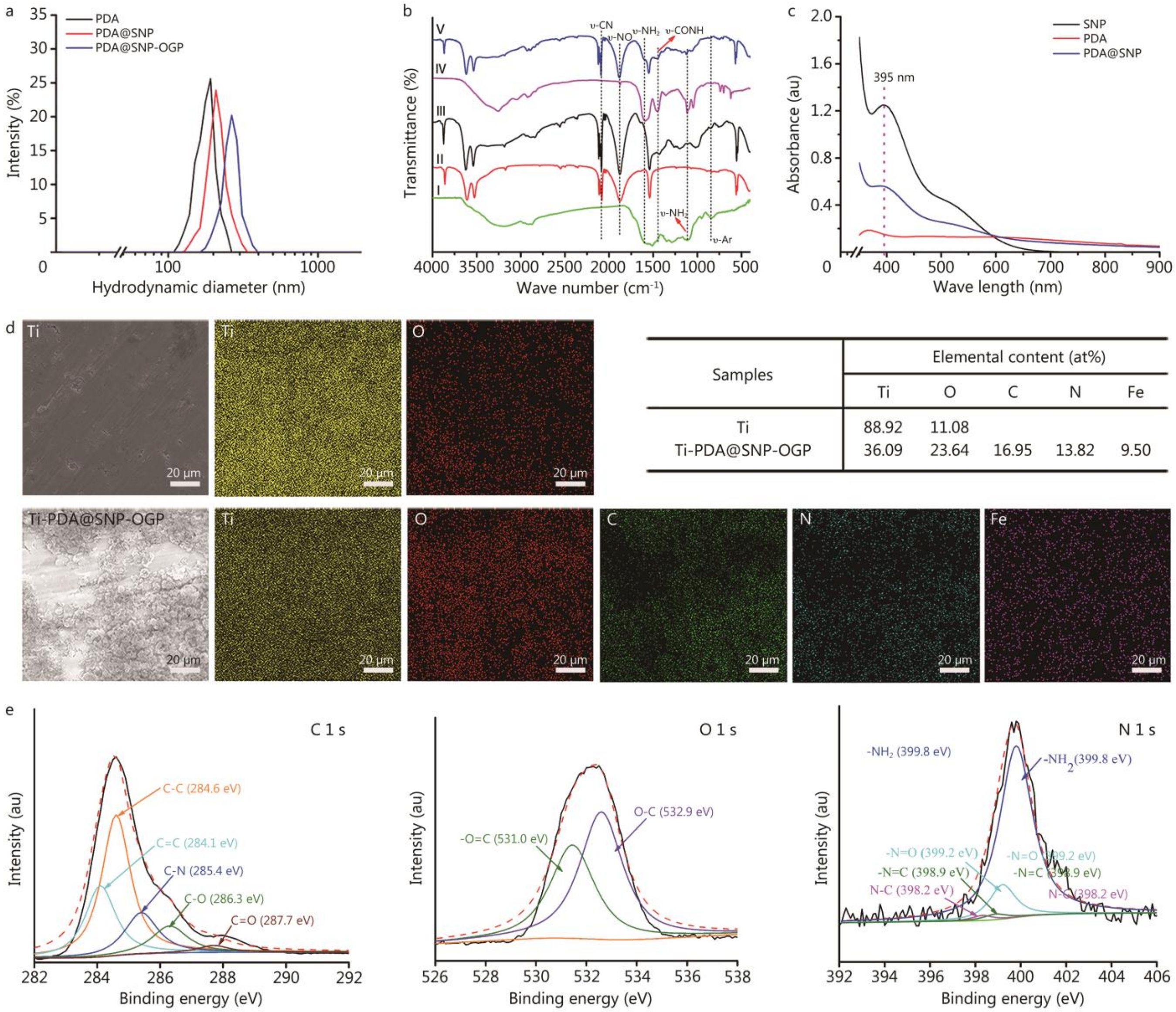

Samples	Elemental content (at%)				
	Ti	O	C	N	Fe
Ti	88.92	11.08			
Ti-PDA@SNP-OGP	36.09	23.64	16.95	13.82	9.50

Fig. S1 Characterization of Ti-PDA@SNP-OGP. **a** Differential light scattering (DLS) measurement of PDA, PDA@SNP, and PDA@SNP-OGP nanoparticles. **b** Fourier Transform infrared spectroscopy (FTIR) of (I) PDA, (II) SNP, (III) PDA@SNP, (IV) OGP, and (V) PDA@SNP-OGP nanoparticles. **c** Ultraviolet-visible (UV-Vis) absorbance of SNP, PDA, and PDA@SNP nanoparticles, and the SNP loading rate of PDA@SNP was calculated based on the standard curve. **d** SEM images of Ti-PDA@SNP-OGP and the corresponding elemental mapping images of Ti, O, C, N, and Fe. Scale bar = 20 μm. **e** X-ray photoelectron spectroscope (XPS) of C 1 s, O 1 s, and N 1 s obtained from Ti-PDA@SNP-OGP. PDA polydopamine nanoparticles, SNP sodium nitroprusside, OGP osteogenic growth peptide, Ti titanium

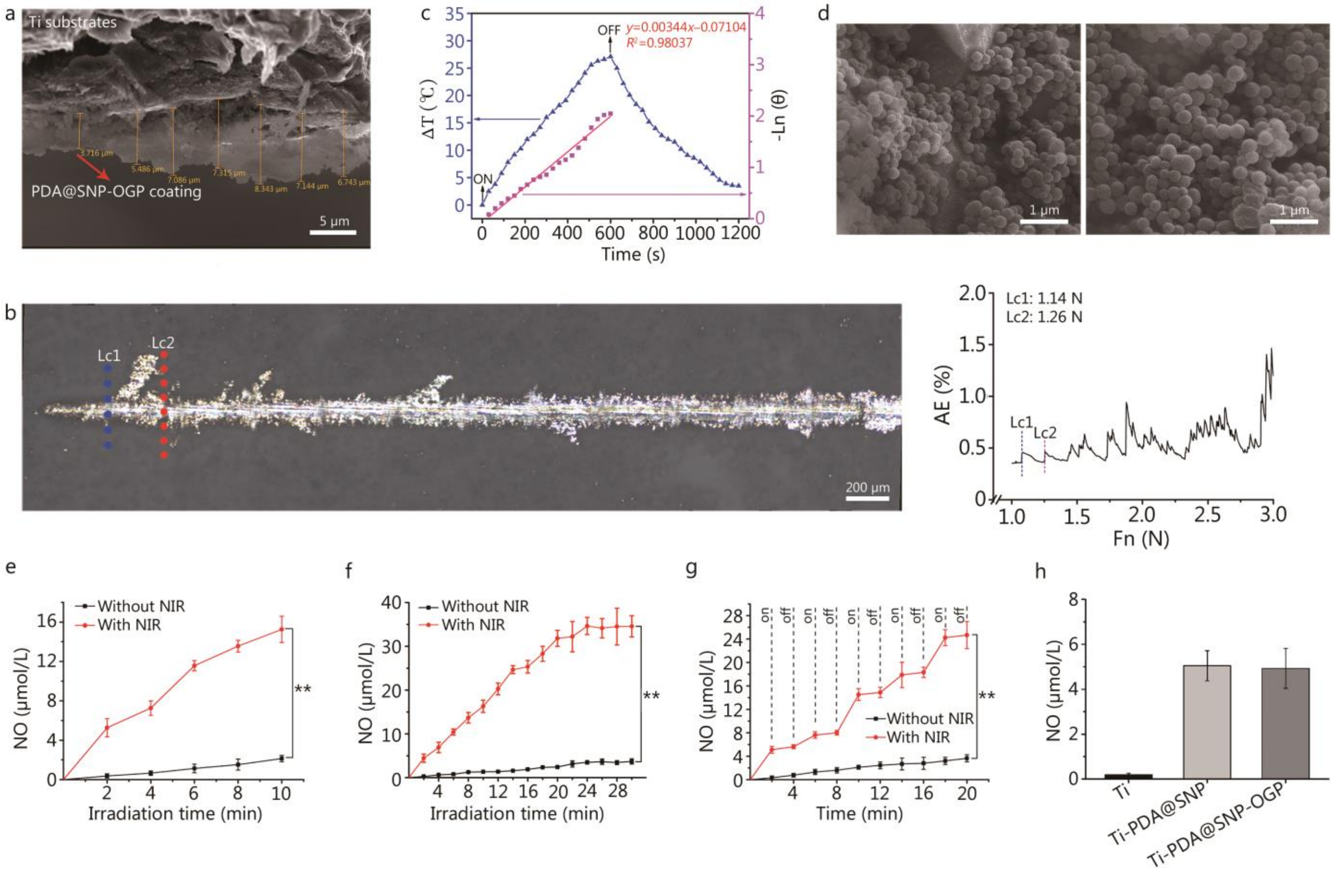

Fig. S2 Characterization of Ti-PDA@SNP-OGP substrate. **a** Cross-sectional scanning electron microscopy (SEM) images. Scale bar = 5 μm. **b** The scratch track images and statistics of critical loads of cohesion (Lc1) and adhesion (Lc2) of Ti-PDA@SNP-OGP. Scale bar = 200 μm. **c** "On-off" temperature change of Ti-PDA@SNP-OGP with NIR irradiation (808 nm, 1.00 W/cm^2) and liner cooling time data vs. –Ln (θ) vs. negative natural logarithm of driving force temperature. **d** The surface morphology changes of Ti-PDA@SNP-OGP before (left) and after (right) NIR irradiation. Scale bar = 1 μm. **e** The cumulative concentrations of NO from Ti-PDA@SNP-OGP with or without NIR irradiation (808 nm, 1.00 W/cm^2) for 10 min. **f** The cumulative concentrations of NO release from Ti-PDA@SNP-OGP with or without NIR irradiation (808 nm, 1.00 W/cm^2) for 30 min. **g** NIR-triggered NO release from Ti-PDA@SNP-OGP with NIR irradiation (808 nm, 1.00 W/cm^2) for 2 min and followed by an interval of 2 min in each cycle. **h** The cumulative concentrations of NO release from different substrates without NIR irradiation after 72 h. $^{**}P < 0.01$; PDA polydopamine nanoparticles, SNP sodium nitroprusside, OGP osteogenic growth peptide, Ti titanium, NIR near-infrared light, NO nitric oxide, AE Acoustic Emission, Fn force normal, Ln logarithm

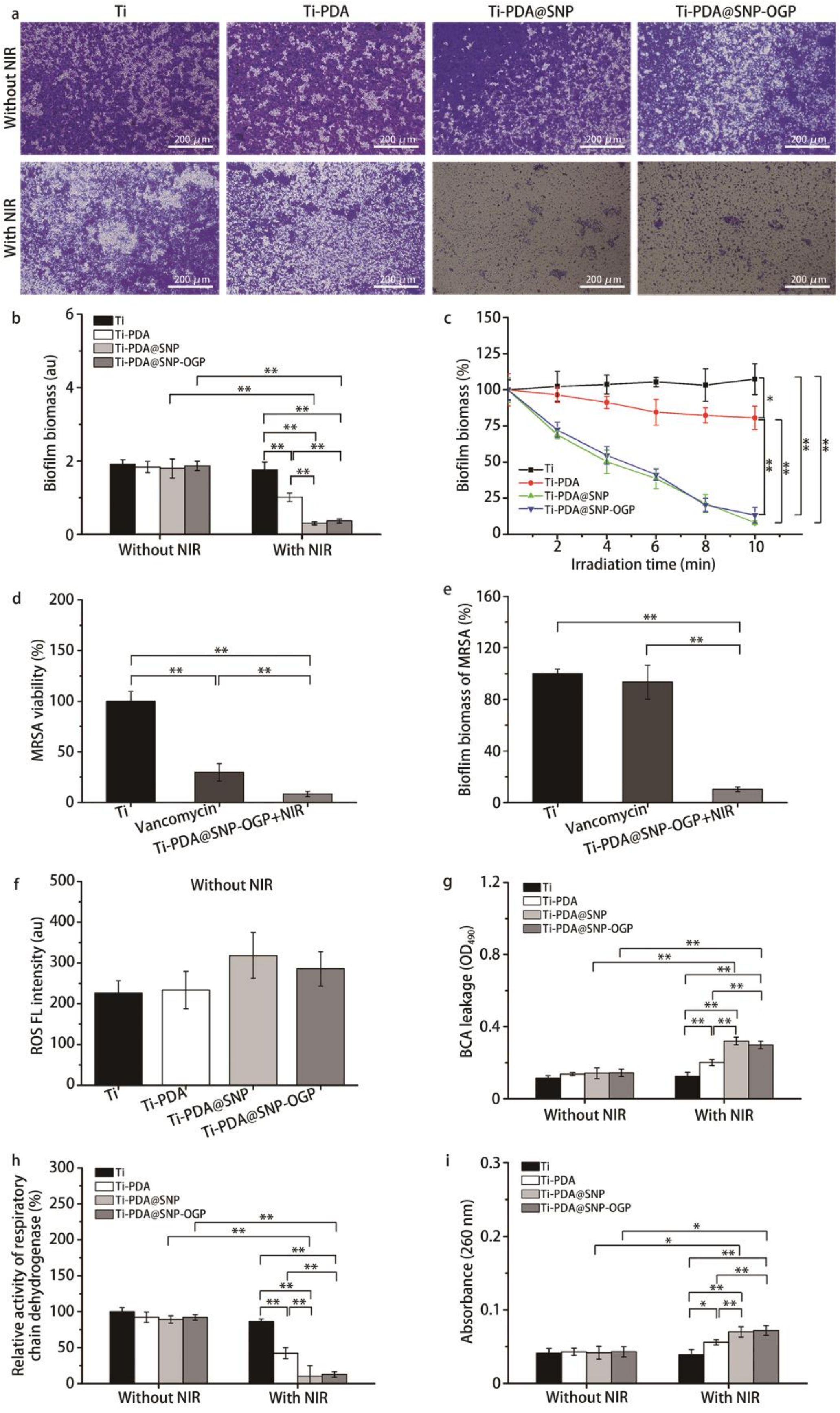

a
Ti
Ti-PDA
Ti-PDA@SNP
Ti-PDA@SNP-OGP
Without NIR
With NIR
200 μm
b
Biofilm biomass (au)
Ti
Ti-PDA
Ti-PDA@SNP
Ti-PDA@SNP-OGP
Without NIR
With NIR
c
Biofilm biomass (%)
Irradiation time (min)
Ti
Ti-PDA
Ti-PDA@SNP
Ti-PDA@SNP-OGP
d
MRSA viability (%)
Ti
Vancomycin
Ti-PDA@SNP-OGP+NIR
e
Bioflim biomass of MRSA (%)
Ti
Vancomycin
Ti-PDA@SNP-OGP+NIR
f
Without NIR
ROS FL intensity (au)
Ti
Ti-PDA
Ti-PDA@SNP
Ti-PDA@SNP-OGP
g
BCA leakage (OD490)
Ti
Ti-PDA
Ti-PDA@SNP
Ti-PDA@SNP-OGP
Without NIR
With NIR
h
Relative activity of respiratory chain dehydrogenase (%)
Ti
Ti-PDA
Ti-PDA@SNP
Ti-PDA@SNP-OGP
Without NIR
With NIR
i
Absorbance (260 nm)
Ti
Ti-PDA
Ti-PDA@SNP
Ti-PDA@SNP-OGP
Without NIR
With NIR

Fig. S3 Mechanism of inhibition on methicillin-resistant *Staphylococcus aureus* (MRSA) biofilms. **a-b** Crystal violet staining of MRSA biofilm in Ti or functionalized Ti substrate with or without NIR irradiation (**a**) and corresponding biomass of MRSA biofilms (**b)** on different substrates. Scale bar = 200 μm. **c** Biomass of MRSA biofilms on different substrates after NIR irradiation (808 nm, 1.00 W/cm^2) for 2, 4, 6, 8 or 10 min. **d** Antibacterial rate of native Ti, Vancomycin or Ti-PDA@SNP-OGP + NIR against MRSA biofilms. **e** Biomass inhibition of native Ti, Vancomycin or Ti-PDA@SNP-OGP + NIR after 2 days of treating the formed biofilm in vitro. **f** ROS FL intensity of MRSA in Ti or functionalized Ti substrate without NIR irradiation. **g** BCA leakage of MRSA in Ti or functionalized Ti substrate with or without NIR irradiation. **h** Relative activity of respiratory chain dehydrogenase of MRSA in Ti or functionalized Ti substrate under various conditions. **i** Leakage of intracellular components from MRSA in different groups with or without NIR irradiation. $^{*}P < 0.05$, $^{**}P < 0.01$; Ti titanium, PDA polydopamine nanoparticles, SNP sodium nitroprusside, OGP osteogenic growth peptide, NIR near-infrared light, ROS reactive oxygen species, FL fluorescence

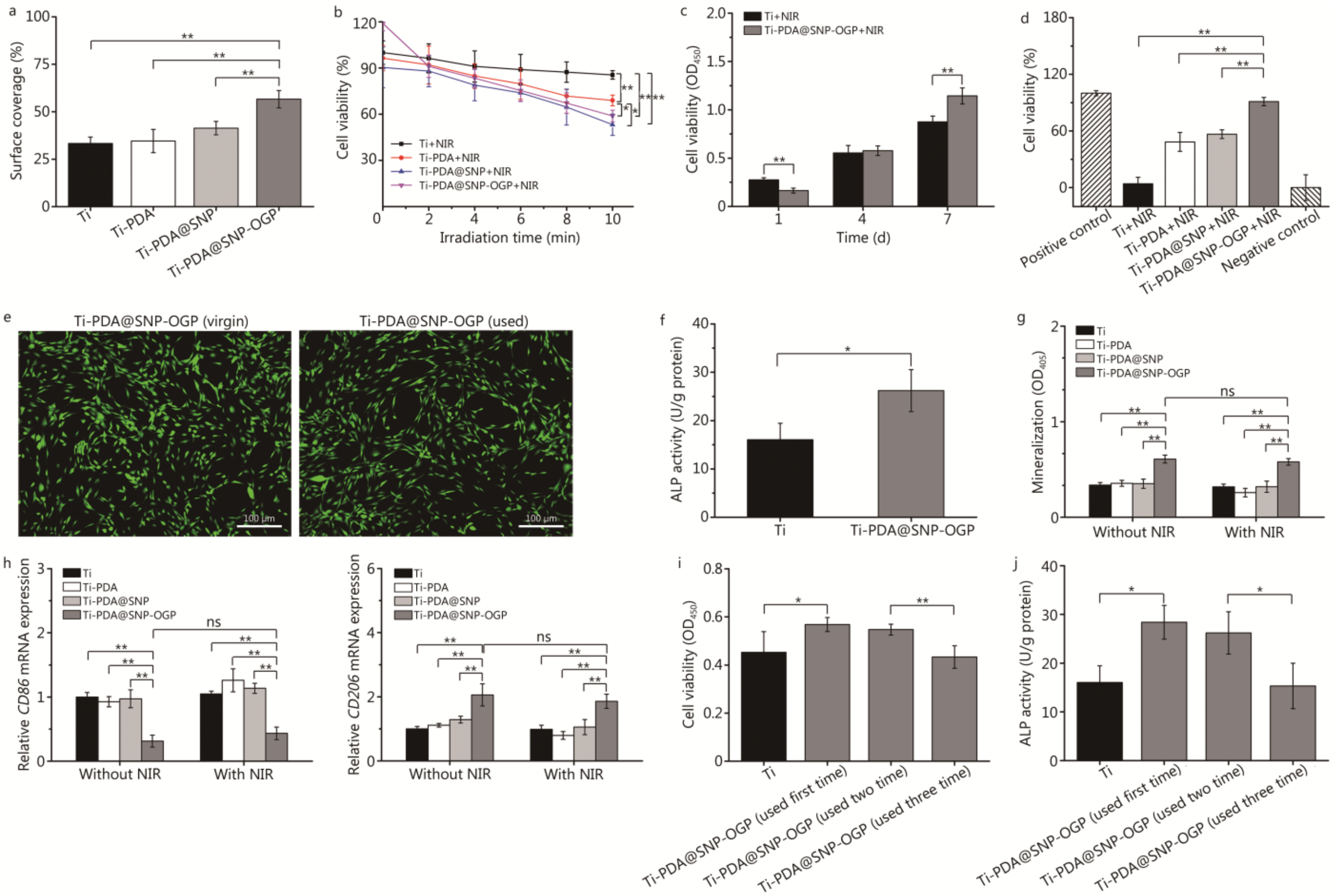

Fig. S4 Biocompatibility, anti-inflammation, and duplication evaluation of Ti or functionalized Ti substrate. **a** Percentage surface coverage of adhering MSCs on different substrates according to the result of fluorescence staining (**Fig. 3a**). **b** Cell viability of MSCs in different groups after NIR irradiation (808 nm, 1 W/cm^2) for 2, 4, 6, 8 or 10 min. **c** Cell viability of MSCs in different groups after NIR irradiation (808 nm, 1 W/cm^2) for 10 min, and further cultured for 1, 4 or 7 d at 37 °C and 5% CO_2. **d** Cell viability of MSCs on Ti or functionalized Ti substrate with NIR irradiation by LDH assay. Positive control group: MSCs cultured on Ti without MSRA. Negative control group: MSCs cultured on Ti in the presence of MRSA without NIR irradiation. **e** Fluorescent staining of MSCs cultured on Ti-PDA@SNP-OGP (virgin) or Ti-PDA@SNP-OGP (used). Scale bar = 100 μm. **f** Alkaline phosphatase activity of Ti-PDA@SNP-OGP group after biofilm eradication. Ti was used as the control. **g** Mineralization of MSCs on Ti or functionalized Ti substrate with or without NIR irradiation by Alizarin red staining. **h** *CD86* and *CD206* expressions of RAW264.7 cells with or without NIR irradiation. **i** Cell viability of MSCs cultured on Ti and Ti-PDA@SNP-OGP after the MRSA biofilm elimination. **j** ALP activities of MSCs on Ti and Ti-PDA@SNP-OGP used for the first, second, and three time after incubation for 7 d. Ti was used as the

control. $^{*}P < 0.05$, $^{**}P < 0.01$; Ti titanium, PDA polydopamine nanoparticles, SNP sodium nitroprusside, OGP osteogenic growth peptide, NIR near-infrared light, CD86 cluster of differentiation 86, CD206 cluster of differentiation 206, ALP alkaline phosphatase

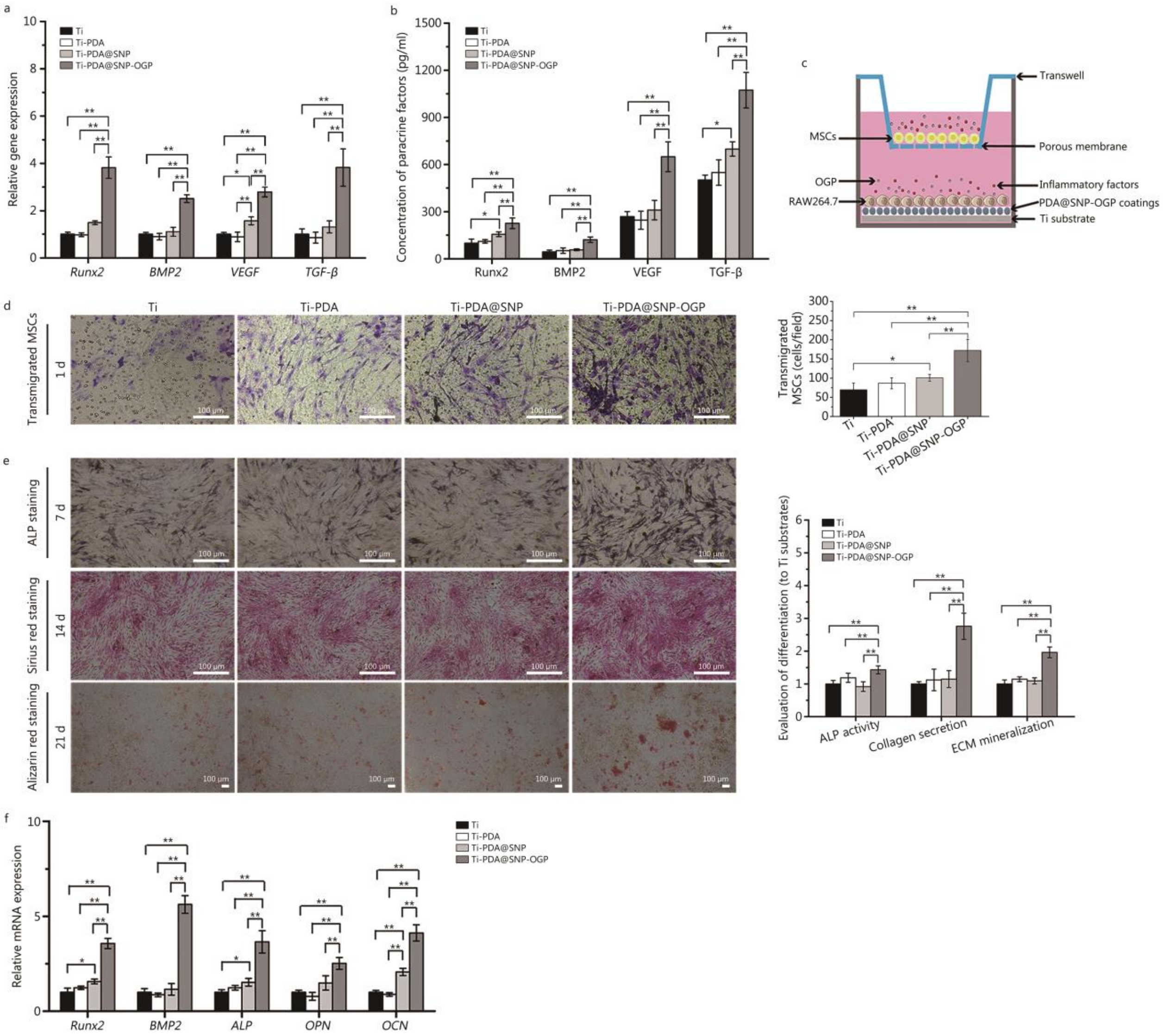

Fig. S5 In vitro osteo-immunomodulation of Ti-PDA@SNP-OGP. **a** mRNA expression of paracrine mediators in RAW264.7 cells. **b** Protein expression of paracrine factors in RAW264.7 cells measured by ELISA. **c** Schematic of MSCs and RAW264.7 cells co-culture system. **d** Representative transmembrane migration staining of MSCs after co-culturing for 1 d. Scale bar = 100 μm. **e** ALP activity, collagen secretion and ECM mineralization. **f** mRNA expression of osteogenesis-related genes of MSCs. $^{*}P < 0.05$, $^{**}P < 0.01$; Ti titanium, PDA polydopamine nanoparticles, SNP sodium nitroprusside, OGP osteogenic growth peptide, ECM extracellular matrix, Runx2 runt-related transcription factor 2, BMP2 bone morphogenetic protein 2, VEGF vascular endothelial growth factor, TGF-β transforming growth factor-β, MSCs bone marrow stromal cells, ALP alkaline phosphatase, OPN osteopontin, OCN osteocalcin

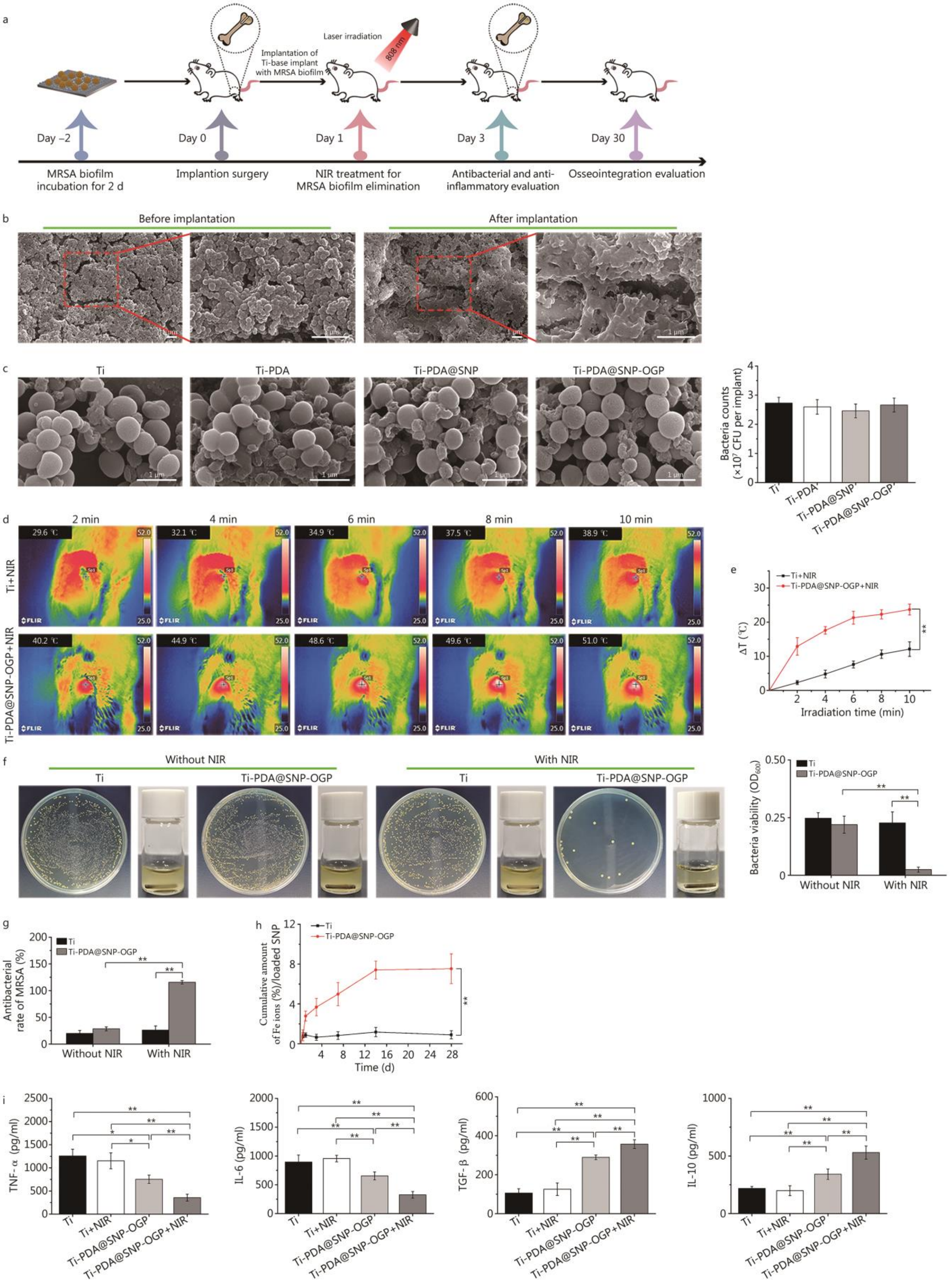

a
Laser irradiation
808 nm
Implantation of Ti-base implant with MRSA biofilm
Day –2
Day 0
Day 1
Day 3
Day 30
MRSA biofilm incubation for 2 d
Implantion surgery
NIR treatment for MRSA biofilm elimination
Antibacterial and anti-inflammatory evaluation
Osseointegration evaluation
b
Before implantation
After implantation
c
Ti
Ti-PDA
Ti-PDA@SNP
Ti-PDA@SNP-OGP
Bacteria counts (×10^7 CFU per implant)
d
2 min
4 min
6 min
8 min
10 min
Ti+NIR
Ti-PDA@SNP-OGP+NIR
29.6 ℃
32.1 ℃
34.9 ℃
37.5 ℃
38.9 ℃
40.2 ℃
44.9 ℃
48.6 ℃
49.6 ℃
51.0 ℃
e
Ti+NIR
Ti-PDA@SNP-OGP+NIR
ΔT (℃)
Irradiation time (min)
f
Without NIR
With NIR
Ti
Ti-PDA@SNP-OGP
Bacteria viability (OD600)
g
Antibacterial rate of MRSA (%)
h
Cumulative amount of Fe ions (%)/loaded SNP
Time (d)
i
TNF-α (pg/ml)
IL-6 (pg/ml)
TGF-β (pg/ml)
IL-10 (pg/ml)
Ti
Ti+NIR
Ti-PDA@SNP-OGP
Ti-PDA@SNP-OGP+NIR

Fig. S6 Antibacterial and anti-inflammatory evaluation in vivo. **a** Schematic of Ti-PDA@SNP-OGP for eradication of MRSA biofilms, inhibition of inflammatory responses and promotion of osseointegration in vivo. **b** SEM images of Ti-PDA@SNP-OGP coating before or after implantation. Scale bar = 1 μm. **c** SEM images of MRSA biofilms formed on different substrates for 2 d and quantitative measurement of bacteria counts. Scale bar = 1 μm. **d** Thermal images of Ti or Ti-PDA@SNP-OGP rods in the tibia. **e** Temperature change (ΔT) curves in Ti or Ti-PDA@SNP-OGP. **f** Antibacterial spread plate images and corresponding antibacterial efficiency of MRSA at day 3 in vivo. **g** Statistical analysis of MHB medium containing Ti or Ti-PDA@SNP-OGP implant. **h** The in vivo distribution and degradation of Ti-PDA@SNP-OGP. **I** In vivo cytokine measurements of surrounding soft tissues and femur bones. $^{*}P < 0.05$, $^{**}P < 0.01$; Ti titanium, PDA polydopamine nanoparticles, SNP sodium nitroprusside, OGP osteogenic growth peptide, CFU colony forming units, OD optical density, NIR near-infrared light, MRSA methicillin-resistant *Staphylococcus aureus*, TNF-α tumor necrosis factor-α, IL-6 interleukin-6, TGF-β transforming growth factor-β, IL-10 interleukin-10, ΔT temperature change

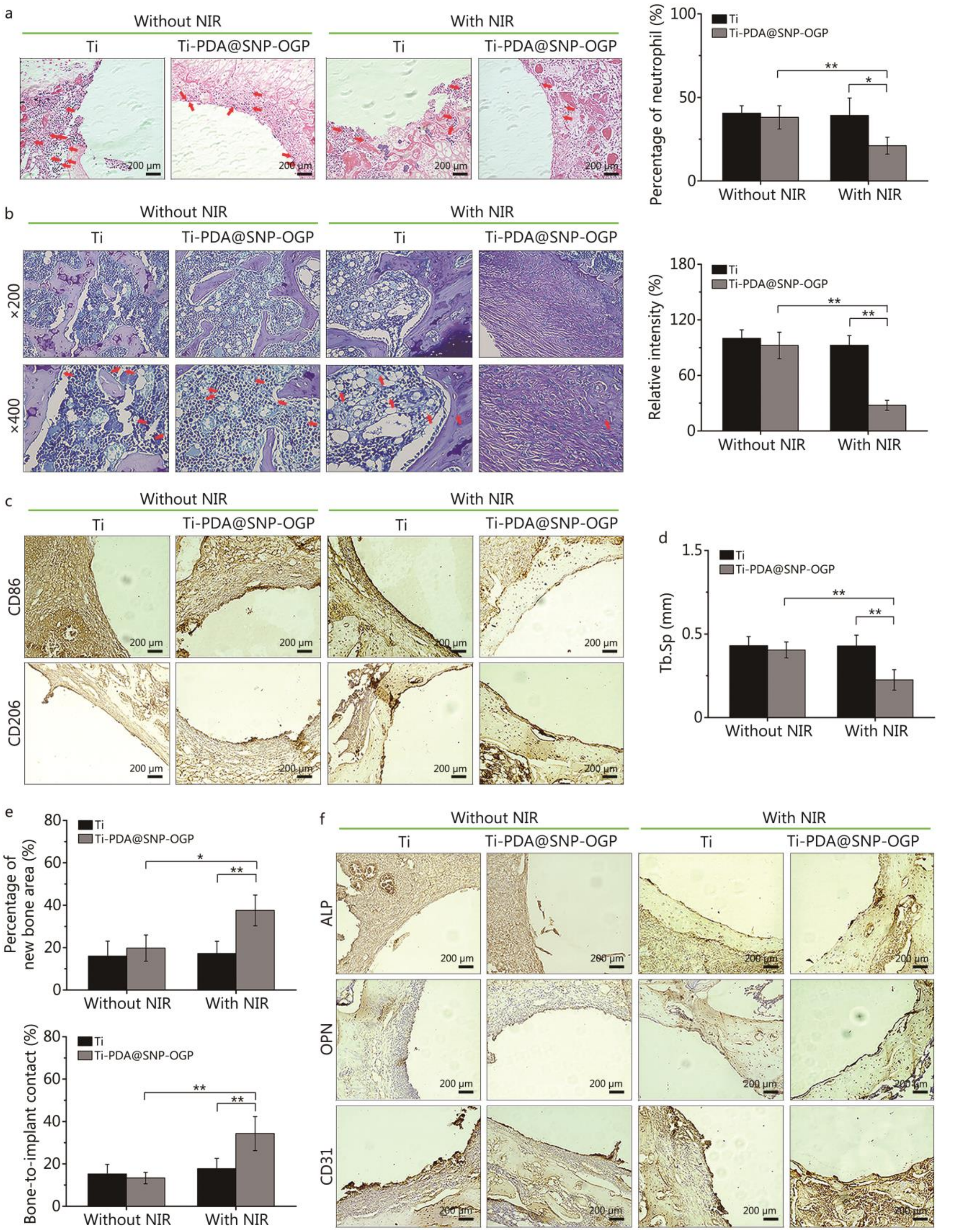

Fig. S7 Anti-inflammation, antibacterial activity and bone regeneration in vivo. **a** HE staining in Ti or Ti-PDA@SNP-OGP group with or without NIR irradiation, and relative quantitative percentage of neutrophils in vivo. Scale bar = 200 μm. **b** Giemsa staining in Ti or Ti-PDA@SNP-OGP group with

or without NIR irradiation, and relative quantitative inflammatory intensity in vivo. Red arrows indicate residual bacteria. Scale bar = 200 μm. **c** Representative CD86 and CD206 IHC staining images at bone-implant interface. **d** Quantitative calculation of trabecular separation (Tb.Sp) based on micro-CT analysis (**Fig. 5a**). **e** Quantitative analysis of percentage of new bone area and bone-to-implant contact of the different groups based on H&E staining (**Fig. 5c**). **f** Representative ALP, OPN and CD31 IHC staining images at bone-implant interface. Scale bar = 200 μm. $^{*}P < 0.05$, $^{**}P < 0.01$; Ti titanium, PDA polydopamine nanoparticles, SNP sodium nitroprusside, OGP osteogenic growth peptide, CD86 cluster of differentiation 86, CD206 cluster of differentiation 206, ALP alkaline phosphatase, OPN osteopontin, CD31 cluster of differentiation 31

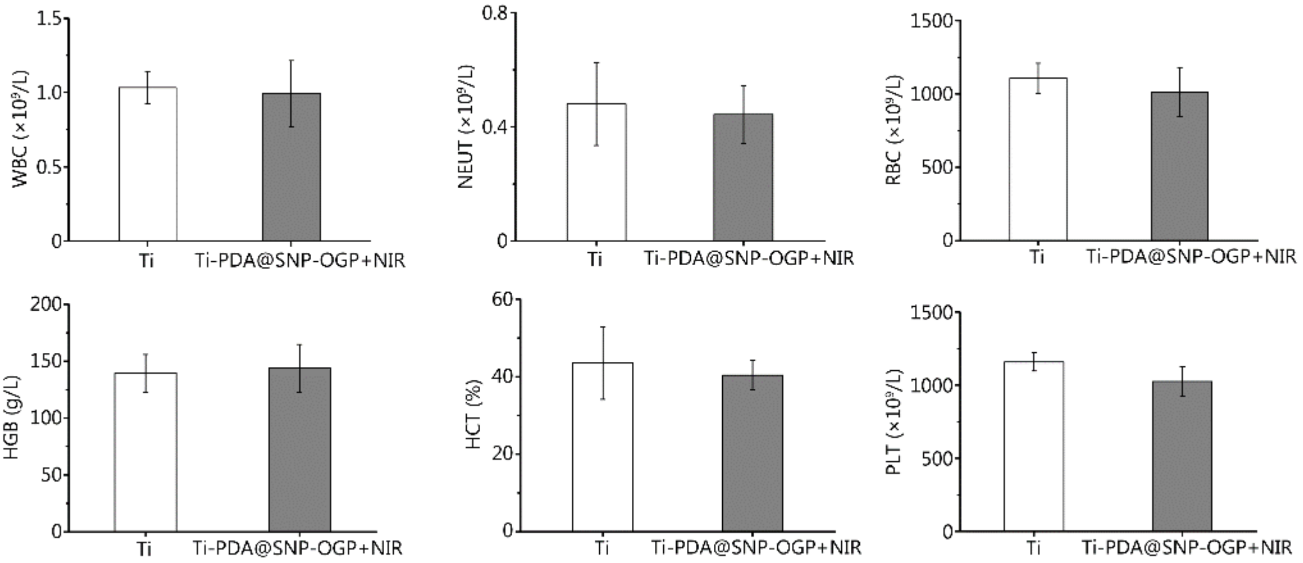

Fig. S8 Biosafety of Ti-PDA@SNP-OGP at the mild temperature (~51 ℃) induced by PTT in vivo. Whole blood biochemical analysis of Ti or Ti-PDA@SNP-OGP + NIR group. WBC white blood cells, NEUT neutrophil granulocytes, RBC red blood cells, HGB hemoglobin, HCT hematocrit, PLT platelets
